# Impact of a Personalized, High-Dose, Intensive Motor Rehabilitation Program, Integrating Advanced Technology for Adults With Central Neurological Conditions (INTeRAcT): Protocol for a Single-Blind Randomized Controlled Trial With a Clinical, Health Economic, and Process Evaluation

**DOI:** 10.2196/93234

**Published:** 2026-05-04

**Authors:** Marjan Coremans, Ingue Allewijn, Filiep Bataillie, Laure De Bruyn, Maaike Fobelets, Femke Jacobs, Sienke Janssens, Laura Pattyn, Koen Putman, Agaat Schiltz, Lisa Tedesco Triccas, Floris Van Thienen, Geert Verheyden

**Affiliations:** 1 Department of Rehabilitation Sciences, KU Leuven, Tervuursevest 101, Leuven, Flanders, 3001, Belgium, +32 16329116; 2 Department of Public Health, Interuniversity Centre for Health Economics Research, Vrije Universiteit Brussel, Brussels, Belgium; 3 Department of Public Health, Research Centre for Digital Medicine, Vrije Universiteit Brussel, Brussels, Belgium; 4 Medical Department, AZ Herentals, Herentals, Flanders, Belgium; 5 Department of Public Health, Faculty of Medicine and Pharmacy, Vrije Universiteit Brussel, Brussels, Belgium; 6 Brussels Institute for Teacher Education, Vrije Universiteit Brussel, Brussels, Belgium; 7 Department of Rehabilitation, To Walk Again, AZ Herentals, Herentals, Flanders, Belgium; 8 Department for Clinical and Movement Neuroscience, University College London, London, England, United Kingdom; 9 Faculty of Rehabilitation Sciences, Hasselt University, Hasselt, Flanders, Belgium; 10 Leuven Brain Institute, KU Leuven, Leuven, Flanders, Belgium

**Keywords:** rehabilitation, high-dose therapy, personalized rehabilitation, stroke, spinal cord injury, chronic phase, randomized controlled trial, clinical trial, health economic evaluation, process evaluation

## Abstract

**Background:**

Chronic stroke and spinal cord injury (SCI) lead to persistent motor impairments that reduce independence and quality of life. Although rehabilitation is essential to address these challenges, the amount of therapy provided during the chronic phase remains limited, while the long-term costs of care are substantial.

**Objective:**

The INTeRAcT (Intensive Rehabilitation Programme Integrating Advanced Technology) trial investigates a high-dose, intensive-targeted, and personalized rehabilitation program through an integrated clinical, health economic, and process evaluation.

**Methods:**

This single-blind randomized controlled trial will include 100 adults in the chronic phase after stroke or SCI. Participants will be randomized to either the INTeRAcT intervention group (n=50) or a control group receiving usual care (n=50). The intervention group will receive 90 hours of personalized motor rehabilitation over 3 weeks, including upper and lower limb therapy, with and without technology, cardiovascular fitness training, and self-management education. Both groups then resume usual care and are followed for 9 months. Clinical assessments are performed at baseline (T0), after 3 weeks (T1, postintervention), and after 9-months follow-up (T2) by a blinded assessor. The primary outcome is independence in daily life, assessed using the Functional Independence Measure for stroke and the Spinal Cord Independence Measure for SCI. Secondary outcomes include the EQ-5D-5L, Canadian Occupational Performance Measure, Goal Attainment Scaling, Fatigue Severity Scale, and stroke-specific measures such as the Action Research Arm Test, Fugl-Meyer Assessment, 6-Minute and 10-Meter Walk-Test, and the Stroke Self-Efficacy Questionnaire. Group differences in clinical change will be analyzed using multivariate linear models. Health economic data will be collected using diaries and questionnaires, capturing direct and indirect costs. Cost-effectiveness will be assessed through a trial-based cost-utility analysis over 9 months and a Markov model over a lifetime horizon. The process evaluation follows the UK Medical Research Council framework, using mixed methods with quantitative and qualitative data from diaries, interviews, and observations, analyzed descriptively and thematically.

**Results:**

The funding of the project started in February 2023. Protocol version 5 (accepted March 15, 2024). Participant recruitment occurred between June 2023 and September 2024, with a total of 102 participants enrolled. Data collection ended in July 2025. Data analysis is ongoing.

**Conclusions:**

This protocol outlines a randomized controlled trial integrating clinical, health economic, and process evaluations to assess a high-dose, individualized rehabilitation program. The findings will provide evidence on effectiveness, cost-effectiveness, and implementation feasibility in chronic stroke and SCI, supporting the optimization of long-term neurorehabilitation care.

## Introduction

Worldwide, 1 in 4 people over the age of 25 years will experience a stroke in their lifetime, resulting in nearly 12 million new strokes each year [1]. In 2021, there were 1.74 million incident stroke cases and 13.4 million prevalent stroke cases reported in Europe [1]. Predictions indicate that by 2030, an additional 3.5% of the adult population will have experienced a stroke [2]. For spinal cord injury (SCI), the incidence in Europe was estimated at 108,000 new cases in 2019, with 2.67 million individuals living with SCI [3].

Both conditions contribute to a significant disease burden [1,4], affect multiple domains of human functioning [5], and are associated with a reduced quality of life [6,7]. Motor problems are the most common impairment [8,9], affecting 60%‐75% of patients after stroke [9] and even more than 80% of individuals with SCI [10], even in the chronic phase [10,11]. Thus, despite completing standard rehabilitation in the subacute phase, deficits remain present in a large number of patients. Motor impairments can involve both the upper and lower limbs, leading to significant limitations in locomotion [12,13] and dependency during daily functioning [11]. To date, there is no cure for poststroke and post-SCI deficits, but rehabilitation can effectively address motor impairments such as reduced strength and balance, as well as functional limitations in performing activities of daily living (ADLs).

In the literature, the so-called “critical window” describes a period of heightened neuroplasticity during which the greatest motor recovery occurs, typically within the first 3 months following a stroke or SCI [14-18]. Around 6 months postonset, motor recovery tends to plateau and generally remains below prepathology function [14-18]. However, Ballester et al [19] demonstrated that an increased sensitivity to rehabilitation can persist beyond 1 year after stroke. A similar phenomenon has been observed in chronic SCI, where several studies have reported motor improvements more than 1 year after onset [20-22]. These findings suggest that patients retain the capacity to enhance their functional abilities through exercise-dependent plasticity [23,24]. Despite this ongoing potential for improvement in the chronic phase, rehabilitation services during this phase remain limited within the current health care system.

Increasing the dose of rehabilitation provides beneficial effects, such as greater improvement in motor ability, reduced activity limitations, and enhanced functional performance [25-27]. Similarly, the integration of advanced rehabilitation technology with conventional therapy has proven effective in improving motor function [28-31] and promoting motor learning [32,33]. In this context, Ward et al [24] provided an intensive rehabilitation program for the upper limb, combining patient-centered therapy with repetitive movements using robotic devices, for individuals with chronic stroke. The program totaled 90 hours, delivered over 3 weeks with 6 hours of therapy per day, 5 days a week. A total of 224 patients completed the program, resulting in significant and clinically meaningful improvements in upper-limb impairment and activity, which were maintained at 6-month follow-up. Although intensive rehabilitation programs often yield positive results, they still present methodological limitations, such as the absence of a control group and the focus on a single body region or therapy component (eg, upper limb rehabilitation or isolated use of technology) [24,26,34]. Building on the study of Ward et al [24], who demonstrated the effectiveness of high-dose upper limb rehabilitation, we will implement a high-dose, intensive-targeted motor rehabilitation program for both the upper and lower limbs. This 90-hour program integrates conventional physiotherapy with advanced technology, cardiovascular fitness training, patient-centered rehabilitation, and self-management education.

To make this level of intensity feasible and effective, advanced rehabilitation technologies will be used. Beyond their demonstrated effectiveness in improving motor function [28-31] and quality of life [35], these technologies offer several advantages. They allow for a greater number of repetitions within a given time frame without overburdening therapists [36], enable the safe progression of intensity [37], and promote patient engagement through gamification, which enhances motivation and supports adherence to recommended therapy doses [38]. These advantages are particularly relevant for chronic stroke and SCI patients, for whom the perception of limited therapy tolerance has been disproven by studies showing successful completion of 300 hours of upper limb therapy over 12 weeks [34,39].

Cardiovascular fitness training is both safe and effective for improving aerobic capacity [40,41]. It plays a crucial role in promoting long-term health and enhancing the quality of life in individuals with SCI [42-45] and stroke [40,41,46-48]. In addition to improving physical fitness, regular cardiovascular exercise helps prevent cardiovascular disease [49,50] and serves as a strategy for secondary stroke prevention [51].

Person-centered goal setting is a fundamental aspect of neurological rehabilitation, yet it remains underused [52,53]. When applied effectively, it enhances a patient’s self-confidence, motivation, treatment adherence, and engagement in rehabilitation [53-55]. Evidence suggests that involving patients in goal setting increases satisfaction with their rehabilitation experience and the achievement of personally meaningful outcomes [56]. Additionally, goal achievement has been associated with improved self-efficacy and more positive perceptions of participation in daily and community life [57]. These benefits contribute to a more rewarding rehabilitation process, promoting long-term success.

Lastly, a self-management program is included to equip participants with the knowledge and skills to manage their symptoms and the impact of their condition on daily functioning [58]. Self-management has been shown to reduce the negative consequences of chronic conditions and to improve overall health and quality of life [59]. Strengthening self-management skills may also positively influence rehabilitation outcomes, functional independence, and participation [60,61].

Therefore, the primary aim of this study is to conduct a phase III clinical trial, including a high-quality randomized controlled trial, to evaluate the clinical effectiveness of this novel motor rehabilitation package.

One of the main cost drivers in long-term health care is the level of disability [62,63]. In addition, the increasing incidence and prevalence of stroke and SCI, driven by different underlying reasons such as increased exposure to risk factors, improved survival rates, and shifting demographic trends, will substantially increase the economic burden of these conditions across Europe [3,64-68]. This translates into increased expenditures for both health care and social services [69,70]. For stroke, the total cost of ongoing care is estimated at €45 to €60 billion annually [64] and is expected to rise further over the next decade [71]. Although less prevalent, SCI also imposes a considerable financial burden, with estimated annual costs ranging from €92 to €212 million [70]. Neurorehabilitation is resource-intensive; therefore, it is essential to consider the potential trade-offs between relevant costs and effects of rehabilitation care.

However, a persistent knowledge gap between clinicians, health economists, and policymakers hampers the development and implementation of innovative rehabilitation approaches. This is partly due to the limited, inconsistent, or inconclusive nature of existing studies [72] in the emerging field of “rehabilitation economics.” While several studies have suggested that increased rehabilitation intensity in stroke may be beneficial, the overall evidence remains inconclusive [73]. For SCI, economic evaluations have primarily focused on acute rehabilitation or locomotor-specific interventions [74-76]. Moreover, few studies have investigated the cost-effectiveness of advanced technology-based rehabilitation [72]. Although some interventions demonstrated cost-effectiveness or reductions in health care usage [77-80], most studies were limited by too short follow-up periods [79] or by the use of a single technological device [77,79,81].

To accurately evaluate the cost-effectiveness of new rehabilitation approaches in the chronic phase, there is a need for clinical trials conducted in outpatient settings [80,82], as well as economic evaluations performed from both the health care system and societal perspectives [83]. Additionally, using microlevel data, where all resources uzed by each participant are directly recorded, can significantly enhance the accuracy and reliability of cost estimations [84,85].

Therefore, a second aim of this study is to conduct a health economic evaluation alongside the INTeRAcT (Intensive Rehabilitation Programme Integrating Advanced Technology) clinical trial, both to contribute to the existing evidence and to support policymakers in making informed decisions regarding the clinical implementation and potential reimbursement of our INTeRAcT program.

Given the complexity of rehabilitation interventions, multicriteria decision analysis may facilitate decision-making by incorporating a broader spectrum of evaluation criteria beyond traditional cost-effectiveness and clinical efficacy metrics. These may include stakeholder experiences (eg, patients and therapists), contextual factors, feasibility, reproducibility, and long-term sustainability [86]. In parallel, the implementation of novel, evidence-based interventions can be associated with numerous challenges [87]. Consequently, a process evaluation is considered essential when evaluating complex interventions [88,89], as it provides critical insights into implementation quality and fidelity, helps to uncover causal mechanisms, and identifies contextual influences that may explain variations in outcomes [90,91]. Additionally, such evaluations are valuable in understanding why interventions may fail or yield unintended consequences, and in identifying ways to optimize them [90,91].

For instance, in the Queen Square program by Ward et al [24], the process evaluation highlighted that the psychosocial aspects of the intervention were perceived as equally important as the intensity and behavioral training by survivors of stroke, their caregivers, and clinicians [92].

Hence, the third objective of this study is to conduct a process evaluation of the INTeRAcT program, to inform and support its implementation into clinical practice and health policy.

In summary, the overall aim of this randomized, single-blind controlled trial is to evaluate the clinical effectiveness and cost-effectiveness of the INTeRAcT program, a high-dose intensive-targeted 90-hour motor rehabilitation program integrating advanced technology, in adults with stroke or SCI, alongside conducting a process evaluation.

We hypothesize that the INTeRAcT program will (1) lead to significantly greater improvements in functional independence at both immediately postintervention and nine months postbaseline; (2) result in clinically relevant improvements in patient-relevant goals, fatigue, and quality of life; (3) yield significantly greater improvements on the stroke-specific outcome measures; and (4) be cost-effective compared to usual care. In addition, the process evaluation will provide valuable insights to inform and guide future implementation of the INTeRAcT program in daily clinical practice.

## Methods

### Study Design

**Figure 1.**
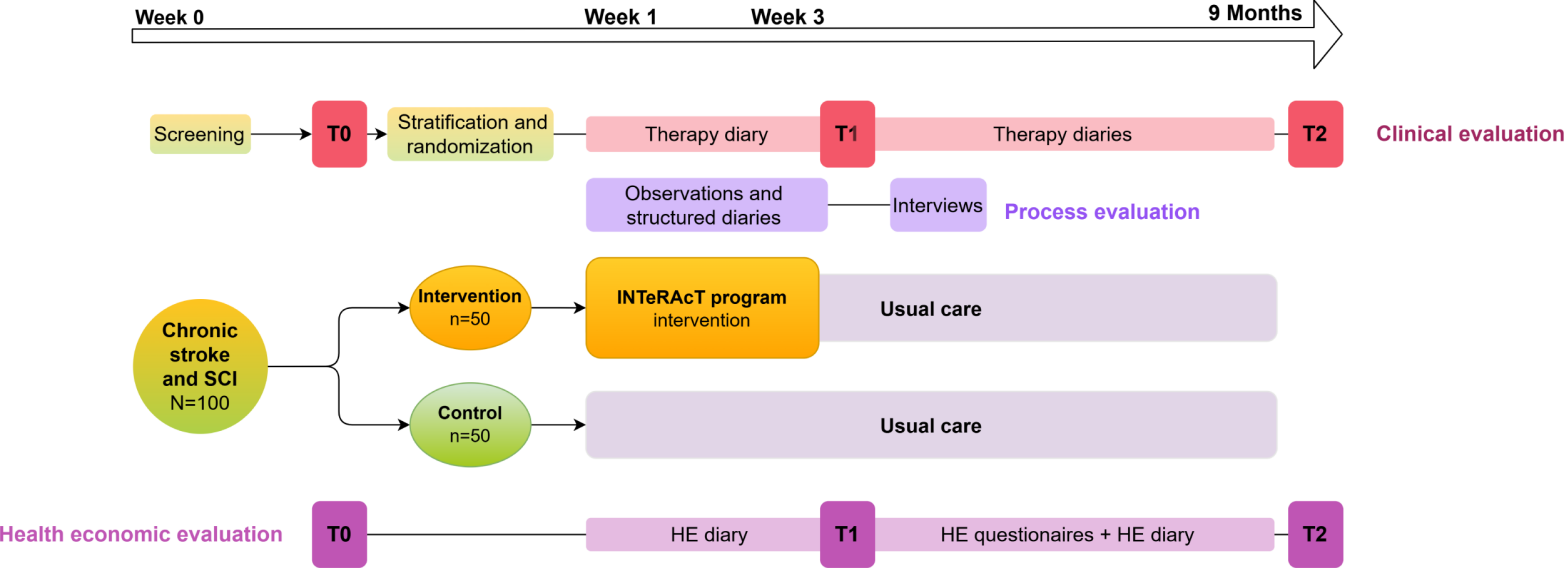
The INTeRAcT study design: the study includes outcome assessments at 3 time points (T0, T1, and T2), a 3-week high-dose rehabilitation program for the intervention group, and a 9-month follow-up period. In parallel with the clinical evaluation, both a process evaluation and a health economic evaluation are conducted. T0, T1, and T2 measurement time points: baseline, after 3 weeks (postintervention), and after 9 months (follow‑up); HE: health economic; SCI: spinal cord injury.

The INTeRAcT trial is an assessor-blinded, randomized, phase III controlled trial (Figure 1). A total of 100 participants with stroke or SCI will be randomly assigned to either a 3-week rehabilitation program (INTeRAcT, n=50) or a control group receiving usual care (n=50). Participants will be stratified for condition and earlier rehabilitation experience with technology. Following the initial 3 weeks, both groups will continue their usual care. All participants will be assessed by a blinded assessor at 3 time points, before the intervention (T0, baseline), after 3 weeks (T1, postintervention), and 9 months postbaseline (T2, follow‑up); and will be followed up for 9 months after baseline. Parallel to the clinical study, a process evaluation and a health economic evaluation are conducted.

### Participant Selection and Recruitment

In this randomized controlled trial, adults in the chronic phase after a stroke or SCI, living in Belgium, participated. Recruitment was conducted via databases (Stroke Rehabilitation Research Team, KU Leuven/To Walk Again), information sessions in hospitals and rehabilitation centers, and outreach to participant associations and adaptive sports centers in Belgium. First-line health care providers, including general practitioners, physiotherapists, speech therapists, home care nurses, and pharmacies, were encouraged to inform potential participants.

The inclusion criteria were (1) first stroke or SCI (American Spinal Injury Association Impairment Scale: A, B, C or D; (A) complete: no sensory or motor function is preserved in sacral segments S4-S5; (B) incomplete: sensory, but not motor, function is preserved below the neurologic level and extends through sacral segments S4-S5; (C) incomplete: motor function is preserved below the neurologic level, and most key muscles below the neurologic level have a muscle grade of less than 3; and (D) incomplete: motor function is preserved below the neurologic level, and most key muscles below the neurologic level have a muscle grade that is greater than or equal to 3) with a clear participant need related to the upper and/or lower extremities, diagnosed by a medical specialist more than three months ago; (2) living at home and more than three months postdischarge from a hospital or rehabilitation center; (3) a maximum recovery of 85% independence in daily functioning, assessed using the Functional Independence Measure (FIM) for stroke or the Spinal Cord Independence Measure (SCIM) for SCI; (4) a prepathology Barthel Index >85/100; (5) unilateral or bilateral weakness of the affected upper and/or lower limbs, with no maximum score of five on all three segments of the Motricity Index; (6) age ≥18 years; and (7) clinical and cognitive ability to perform high-dose rehabilitation with advanced technology, as assessed by a medical doctor (MD).

**Figure 2.**
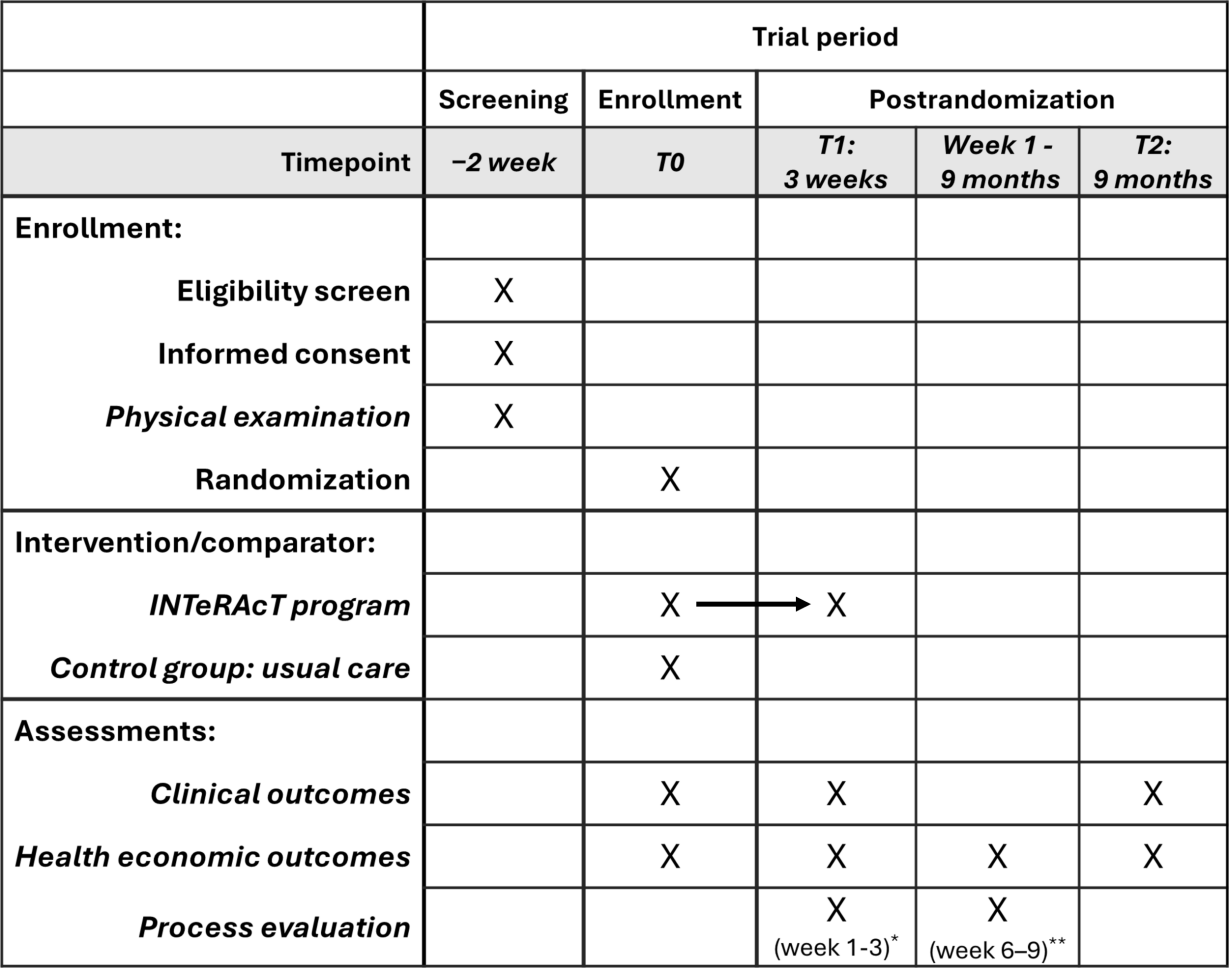
Participant timeline (SPIRIT figure): schedule of enrolllment, interventions, and assessments. T0, T1, and T2: measurement time points: baseline, after 3 weeks (postintervention), and after 9 months (follow‑up); *Weeks 1-3: structured diaries and observations; **Weeks 6-9: semistructured interviews.

Exclusion criteria were (1) no voluntary movements against gravity in the upper and lower limbs; (2) other neurological or musculoskeletal conditions that could interfere with protocol adherence, as assessed by an MD; (3) severe communication, cognitive, language, or visual impairments preventing participation in the intervention or assessment process, as assessed by an MD; (4) any condition that could compromise participant safety or compliance with the clinical investigation plan, as determined by the investigator and Mensure that the entire section on inclusion criteria is consistentD; and (5) pregnancy or breastfeeding. Trial information was provided, and informed consent (Multimedia Appendix 1) was obtained by a researcher of KU Leuven before screening. The screening was conducted by a researcher from KU Leuven in collaboration with a rehabilitation physician from AZ Herentals. A schedule of enrollment, intervention, and assessments can be found in Figure 2.

### Sample Size Calculation

The power calculation was performed based on the primary research question: the evaluation of the short-term clinical effect of the INTeRAcT intervention versus usual care at home on independence in daily living, measured using the FIM/SCIM (see the Clinical Evaluation section). As no previous studies have assessed functional independence in both stroke and SCI participants in the chronic phase as a combined group, the calculation was based on a relevant improvement for this mixed population. A total sample size of 90 participants will provide 80% power to detect a 12% significant and relevant difference [93,94] in FIM/SCIM scores between the 2 groups, assuming an SD of 20% [95,96] and a 2-tailed *α* of 0.05. This corresponds to an effect size of 0.6, which is considered a “medium” effect. To account for an expected dropout of 10%, further, 100 participants will be recruited.

### Randomization and Blinding

To ensure the integrity of this study, the following randomization procedures have been implemented. Participants (n=100) are randomly assigned to 1 of 2 groups: the intervention group, “INTeRAcT” (n=50), or the control group, “usual care” (n=50). To achieve a balanced distribution across conditions and prior rehabilitation technology experience, participants are stratified by condition (stroke or SCI) and prior technology experience. The latter refers to previous participation in outpatient rehabilitation involving advanced technological devices at a frequency of at least 1 to 2 sessions per week for a minimum duration of 3 months. Allocation concealment is ensured using preprepared randomization lists with a block size of 2. As participants enter this study, an independent team member responsible for randomization, who is not involved in participant contact or data collection, assigns each participant to the next concealed allocation. Recruiters and assessors have no access to the allocation lists and are unaware of the randomization sequence or block size. To ensure objectivity and minimize bias, outcome assessments are conducted by an assessor blinded to treatment allocation. Group assignment will be revealed to participants after the first measurement (T0). In exceptional cases, early disclosure may occur due to practical arrangements for participation in the 3-week intervention; however, the assessor will remain blinded. Participants are instructed not to disclose their allocation. Physiotherapists, participants after T0, and researchers involved in follow-up, process evaluation, and economic evaluation cannot be blinded.

### Trial Treatment

#### Experimental Intervention: INTeRAcT Program

The intervention group will receive a total of 90 hours of motor therapy over 3 weeks, 30 hours per week, delivered in 6-hour sessions each weekday. Every 3 weeks, 3 participants will undergo the intervention. The therapy will be provided by trained physiotherapists with expertise in neurological disorders at the clinical site, AZ Herentals (To Walk Again).

**Figure 3.**
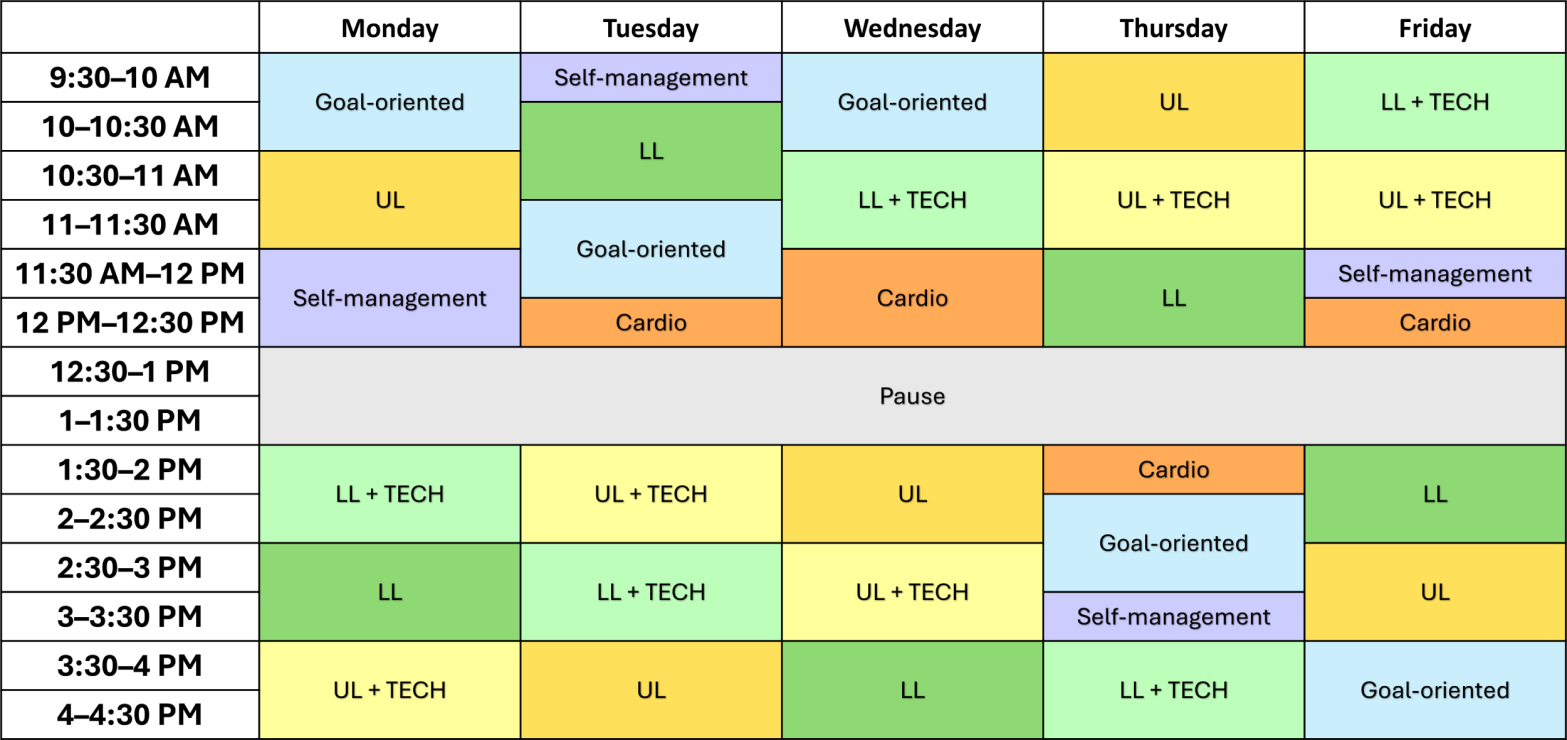
Example of the INTeRAcT program for one week: the therapy program is structured in daily six-hour sessions, comprising the following seven components: (1) UL, (2) LL, (3 and 4) UL/LL + TECH, (5) cardio, (6) goal-oriented therapy, and (7) self-management education. Cardio: cardiovascular fitness training; LL: lower limb therapy; TECH: therapy with technological devices for the upper or lower limb; UL: upper limb therapy.

This high-dose, intensive-targeted rehabilitation program is evidence-based, grounded in the principles of motor learning, personalized, and goal-oriented. The program aims to enhance physical fitness and independence in daily life through cardiovascular fitness training, therapy for the upper and lower limbs, with and without advanced technological devices, and goal-oriented training. All of this is tailored to the individual participant, considering personal goals and limitations, incorporating built-in progression over time. Additionally, the program emphasizes transferring skills to functional tasks and promoting self-management to improve the participant’s self-efficacy beyond the duration of the program. These components constitute the 7 therapy blocks of the program. All blocks are covered daily in a random order, except cardiovascular fitness training and self-management. Figure 3 illustrates an example of a weekly schedule that outlines the various therapy components.

During the baseline (T0) assessment, 3 specific goals will be defined by the participant, in collaboration with a KU Leuven researcher with a master’s degree in neurological physiotherapy, using the Goal Attainment Scaling (GAS) [97,98], which will serve as the focus throughout the 3-week intervention. Based on these goals, the physiotherapist will evaluate which subgoals are still needed to achieve the goal. The overall structure of the program follows a four-step process: (1) setting personalized goals; (2) evaluating and observing the activity to identify the subgoals contributing to the main problem; (3) practicing these subgoals to address underlying difficulties during components 1‐5, as outlined below; and (4) practicing the full activity during the goal-oriented therapy sessions (component 6), as outlined below.

Upper and lower limb therapy sessions without technology (components 1 and 2) follow a conventional therapy approach and include a range of techniques such as mobilization and stretching of the affected limb, analytical and coordination exercises, strength training, task-specific training with repetitions, fall prevention, balance training, and functional exercises. These sessions incorporate both proximal and distal components, emphasizing fine manipulation, sensorimotor training, foot placement, analytical movements, gait adaptability, and complex ADLs. Both upper and lower limb therapy components are provided daily, 1 hour each, for a total of 5 hours per week per component.

In addition to conventional upper and lower limb therapy, participants participate in technology-assisted upper and lower limb therapy (components 3 and 4), with 1 hour per day dedicated to each component. For upper limb rehabilitation, the Gloreha (BTL Robotics) focuses on improving distal hand function through passive or active-assisted mobilization of the fingers. Action observation and interactive games are integrated to stimulate arm and hand use while providing arm support. The OmniHi5 (ACP) uses electrical stimulation to facilitate upper limb movement. The HUR fitness equipment is adapted for individuals in wheelchairs for strength training, but can also be used by ambulatory individuals with a standard chair. For lower limb rehabilitation, an exoskeleton gait robot (Ekso Bionics) assists individuals with limited or no walking ability, whereas the ZeroG Gait & Balance System (Aretech) provides dynamic body weight support in a fall-safe environment to facilitate gait training. The RehaMove (Hasomed), a functional electrical stimulation bike, is used to activate muscles and promote movement. Additionally, the DIERS 4Dmotion Lab integrates a treadmill with a feedback system to improve weight shifting and promote a more normal gait pattern. To complement these technologies, the D-WALL Elite (TecnoBody) offers a versatile platform for designing personalized exercises and managing a broad range of programs focused on posture, functional training, balance, and strength. These advanced technologies are integrated into therapy to align with the participant’s individual rehabilitation goals.

The cardiovascular fitness training [99-101] (component 5) focuses on interval training, using a variety of methods such as a bicycle or arm ergometer, stepping, and Tabata (eg, boxing, circuit training, and ropes). The training intensity is primarily monitored using heart rate. In individuals with complete or incomplete SCI, where muscle mass is limited, the intensity is guided by the Borg Rating of Perceived Exertion Scale [102], aiming to achieve a score of at least 12 of 20. This training is delivered in a group setting for a total of 2.5 hours per week, consisting of one 1-hour session and three 0.5-hour sessions.

During the goal-oriented training session [103] (component 6), the therapist and participant collaboratively design and execute an individualized therapy plan based on the three specific goals previously established. These goals follow the SMART (Specific, Measurable, Achievable, Relevant, and Time-Bound) principles. For example, a goal might be: “After three weeks of training, I want to be able to walk 2 km with my dog.” This approach ensures that therapy is tailored to the participant’s needs and integrated into a highly functional, real-life context. The goal-oriented training is conducted daily, with 1 hour of individual sessions each day, amounting to a total of 5 hours per week.

The self-management sessions (component 7) are based on key principles that emphasize a personalized and flexible approach. It tailors support to individual needs and circumstances, allowing for initiation at any stage. The approach prioritizes the person’s story and promotes supportive relationships while encouraging autonomy. Emphasis is placed on small, meaningful actions in daily life, reinforcing autonomy and motivation. It builds on individuals’ existing self-management strategies and social support systems to enhance long-term engagement and adaptability [104]. Self-management sessions comprise a total of 2.5 hours per week, including 1 hour of group session and 3 individual sessions of 0.5 hours each. Group therapy allows participants to learn from each other and exchange experiences and strategies, while individual sessions provide personalized counseling tailored to each participant’s needs and goals.

During the 3-week intervention period, the intensive therapy is documented daily in diaries by the physiotherapists, including the total number of minutes of therapy, the division between active therapy time (eg, performing active repetitive movements) and other therapy time (eg, explanation, resting periods, and ADL tasks), and the content of each of the 7 therapy blocks. Additionally, at the end of each therapy block, the Borg Rating of Perceived Exertion Scale [105] is completed to assess participants’ subjective experiences of physical exertion. This scale ranges from 6 (“no exertion at all”) to 20 (“maximal exertion”) and allows for individualized adjustments to training intensity. Outside the 6-hour daily intervention sessions, participants were permitted to continue their usual care, which was recorded in diaries. The intervention was described according to the TIDieR (Template for Intervention Description and Replication) checklist, provided in Checklist 1.

#### Control Intervention: Usual Care

To allow a pragmatic comparison with current clinical practice, the control group will continue their usual care at home or in an outpatient rehabilitation center or hospital for the entire 9-month study period, without receiving any alternative interventions. After completing this study, participants in the control group will be given the opportunity to receive the identical 3-week program following the final follow-up measurement at 9 months. This waitlist-control design will help maintain participants’ motivation to adhere to this study during the 9-month follow-up period.

Participants will document their usual care in therapy diaries (Figure 1), with assistance from their therapist if needed. These diaries will capture the frequency (number of sessions), intensity, and time (duration), and type (therapy content) of physiotherapy, occupational therapy, and psychological sessions, in line with the FITT (Frequency, Intensity, Time, and Type) principles [101]. The diaries were developed by the research team based on expert input, and their content was reviewed and approved by clinicians. Participants in the intervention group will complete the same diaries during the follow-up period, after the 3-week intervention. The diaries will be collected and reviewed monthly, and any missing data will be requested from participants.

### Adverse Events

The risk of adverse events (AEs) occurring during this study is considered low; therefore, safety reporting will be limited to the safety reporting that is necessary in routine care. Participants will be asked to report any AE related to this study-specific intervention to this study’s team. This study’s team will keep detailed records of all reported AEs, which will be assessed by the investigator at the rehabilitation site, an MD, regarding their seriousness, causality, and expectedness. A structured procedure has been established to ensure proper reporting of AEs to the relevant regulatory committees. If an AE directly or indirectly related to study participation requires additional medical care, the associated costs will be covered by this study’s insurance.

The following events are commonly observed and are therefore not considered as AEs for this study: (1) during or after the treatment, the participant may feel the following symptoms or events: fatigue, muscle spasm or increase in muscle tone and soreness, as with any movement exercise with a device that activates the body and muscles. These types of symptoms are anticipated and should fade out; (2) redness of the skin, light bruising, or small superficial pressure injuries where the equipment has exerted pressure on the skin, from harnesses or use of the revalidation robots. These injuries will be subject to follow-up as they are in usual care: close monitoring by the therapist and use of pressure bandages to prevent them; (3) orthostatic hypotension: blood pressure drop due to position change (usually due to verticalizing: sitting or lying to standing); and (4) changes in bladder and bowel control after activation of the participant.

Premature discontinuation or interruption of the intervention may occur due to AEs, protocol violations, pregnancy, or illness. In such cases, clinical follow-up will be ensured, and if illness interferes with therapy, continuation or restarting the intervention will be considered where feasible.

### Participant and Public Involvement

Survivors of stroke and SCI reviewed and provided input on the various study documents that participants are required to complete during this study. Adjustments were made based on their feedback. A scientific committee composed of clinical stakeholders and researchers has been established and will be involved throughout the entire course of this study.

### Outcomes

#### Clinical Evaluation

The primary objective of this study is to evaluate the clinical benefits of the INTeRAcT program across various domains of the International Classification of Functioning, Disability, and Health (ICF) immediately postintervention and after 9 months of follow-up [106]. Therefore, participants are assessed by a blinded assessor at 3 different time points: T0, T1, and T2 (Figure 1). The assessor remains blinded to treatment allocation to ensure objective outcome evaluation. Participants will be reminded of their upcoming assessment appointments promptly via email or telephone. During data collection, a standardized case report form is completed. This form includes researchers’ notes, demographic and pathology-related data, and the results of the standardized clinical assessments and questionnaires.

##### Data: Clinical Outcome Measures

**Figure 4.**
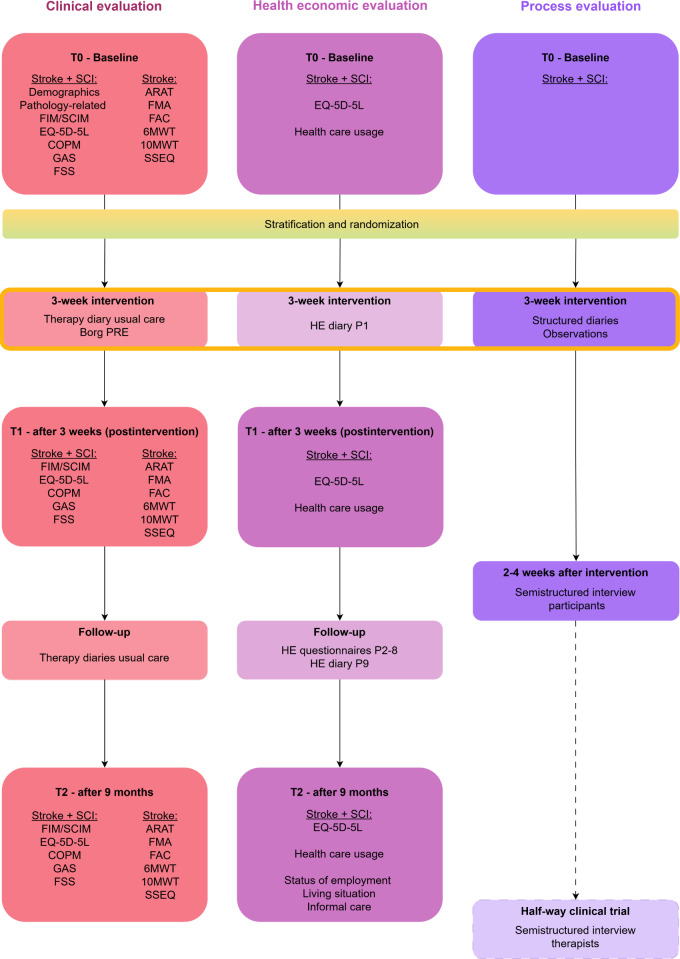
Flowchart for data collection of the INTeRAcT trial. The figure outlines the timing of clinical, health economic, and process evaluations for participants throughout this study. The “halfway clinical trial” section indicates data collection of the interviews with the INTeRAcT therapists. 6MWT: 6-minute walk test; 10MWT: 10-meter walk test; ARAT: Action Research Arm Test; BORG PRE: Borg Rating of Perceived Exertion Scale; COPM: Canadian Occupational Performance Measure; FAC: functional ambulation category; FIM: Functional Independence Measure; FMA: Fugl-Meyer Assessment; FSS: Fatigue Severity Scale; GAS: Goal Attainment Scaling; HE: health economic ; P1-P9: follow-up period 1-9; SCI: spinal cord injury; SCIM: Spinal Cord Independence Measure; SSEQ: Stroke Self-Efficacy Questionnaire.

All outcome measures are collected by a trained, blinded assessor (Figure 4). At baseline (T0), demographic, socioeconomic, and pathology-specific data will be collected. An overview is provided in Multimedia Appendix 2.

The primary outcome of this study is the change in functional independence from baseline to T1, assessed using the FIM and the SCIM.

The FIM-motor [107] evaluates independence in ADLs in individuals with a stroke. It comprises 13 items that assess the level of assistance required for performing ADLs safely and efficiently. These activities include self-care, bladder and bowel management, transfers, and mobility, and are scored on a 7-point ordinal scale ranging from total assistance to complete independence, with a total score between 13 and 91. The FIM has demonstrated acceptable reliability and validity in individuals with stroke [108,109].

The SCIM [110] assesses independence in daily activities in individuals with an SCI. It consists of 19 items divided into 3 subscales: self-care, respiration and sphincter management, and mobility. The total SCIM scores range from 0 to 100, with 0 indicating total assistance and 100 indicating complete independence. The SCIM has been validated and is a reliable measure for this population [111].

Although FIM (stroke) and SCIM (SCI) are condition-specific instruments, both measure the same overarching construct: independence in daily life [112]. To allow pooled analysis across conditions, we analyze percentage change from baseline rather than raw scores, enabling comparison of proportional improvements in functional independence. Both measures will be administered at T0, T1, and T2.

We include several secondary outcome measures representing different levels of the ICF for both stroke and SCI. All secondary outcomes will be administered at T0, T1, and T2.

The EQ-5D-5L [113] measures health-related quality of life across 5 dimensions: mobility, self-care, daily activities, pain or discomfort, and anxiety or depression. Each dimension has 5 response levels: no problems, slight problems, moderate problems, severe problems, and unable to or extreme problems. A weighted health state index is calculated for individuals ranging from less than 0 (a health state perceived as worse than death) to 1 (representing full health), with higher scores indicating better health utility. Additionally, the questionnaire includes a visual analogue scale, where participants rate their perceived health from 0 (worst imaginable health) to 100 (best imaginable health). This scale applies to both stroke and SCI populations [114,115].

The Canadian Occupational Performance Measure (COPM) [116] is an evidence-based outcome measure designed to capture a participant’s self-perception of performance in everyday living over time, across 3 areas: self-care, productivity, and leisure. The participant provides a score between 0 and 10 for importance, performance, and satisfaction. An average score for performance and satisfaction is calculated across all relevant items. It can be used in both study populations [117,118]. In this study, the COPM will also be used to assess the participants’ needs and establish the goals for the GAS, as described below.

The GAS [97] is an individualized evaluation method scored on an ordinal 5-point scale, capturing a person’s individual treatment goals and the extent to which they are achieved. Each goal is rated from +2 (much more than expected) to −2 (much less than expected), with 0 representing the expected level of achievement. The baseline level is typically set at −1, and goals can be weighted by the participant according to their importance or difficulty. GAS will be used for both stroke and SCI populations [119].

The Fatigue Severity Scale [120] is a 9-item scale, assessing the perceived severity of fatigue symptoms over the past week in various daily situations. It evaluates how fatigue impacts functioning and is scored on a 7-point scale, ranging from “strongly disagree” to “strongly agree,” with a minimum total score of 9 and a maximum of 63, with a higher score indicating more impact on daily life. It applies to both populations [121,122].

For the stroke population, additional secondary outcome measures were added, representing different levels of the ICF. All these secondary outcomes will be administered at T0, T1, and T2.

The Action Research Arm Test [123,124] is an observational measure to assess upper extremity performance, including coordination, dexterity, and functioning. It consists of 19 items, with task performance rated on a 4-point scale, ranging from 0 “no movement” to 3 “movement performed normally,” resulting in a total score between 0 and 57, with higher scores indicating better upper limb performance.

The Fugl-Meyer Assessment [125,126] assesses motor functioning in the upper and lower limbs in poststroke participants. It includes 50 test items, with a 3-point ordinal scale for each (0: unable, 1: partial, and 2: [near] normal), resulting in a score between 0 and 100, where 0 indicates no motor function, and 66 indicates an intact motor function.

The Functional Ambulation Category [127,128], a 6-point functional walking test that evaluates ambulation ability, determining how much human support a participant requires when walking and whether or not they use a personal assistive device. A score of 0 indicates the participant cannot walk or needs help from 2 or more persons, while a score of 5 indicates the participant can walk independently.

The 6-minute Walk Test [129,130] measures functional walking capacity by assessing the maximum distance (meters) a participant can cover in 6 minutes.

The 10 Meter Walk Test [129,131] assesses functional mobility and gait by measuring the time needed to walk 10 meters at a comfortable, self-selected speed. Walking speed (m/s) is then calculated as the average of 3 trials.

The Stroke Self-Efficacy Questionnaire [132] is a 13-item self-report scale, evaluating individuals’ confidence in performing ADLs. Each item is rated on a 10-point scale, where 0 indicates “not confident at all” and 10 indicates “very confident,” resulting in a total score ranging from 0 to 130.

For a subset of participants in the intervention group, we will measure time on task (TOT), defined as “repetitive active movements performed by the participant, in line with motor learning principles, and directly related to the therapy block and personalized goals.” Using a stopwatch, we will time the active moments for each therapy component once per week, across the 3-week intervention period. Data for TOT and the process evaluation will be observed simultaneously. We will clearly distinguish between TOT and “other time.” The latter includes ADLs (eg, toileting and drinking), transfer time not related to the goals, rest periods, and preparation time. Observers, who are not the treating therapists, will be positioned to avoid interfering with the therapy while maintaining sufficient visibility and audibility. A standardized method and overview for each therapy block have been developed to guide classification and timing.

##### Data Analysis

We will perform an intention-to-treat analysis, including all measurements from included participants in this trial, irrespective of whether participants prematurely dropped out. Changes from baseline (T0) in FIM and SCIM scores between the experimental and control groups will be compared using a multivariate linear model with an unstructured covariance matrix, including time and treatment as main effects and a time-by-treatment interaction. The stratification variables “experience using advanced rehabilitation technology” and “pathology” will be included as fixed factors. A sensitivity analysis based on multiple imputation will be conducted if substantial missing data (>5% [133]) are present, to assess the robustness of the results under realistic deviations from the missing-at-random assumption [133]. In addition, the analysis will be repeated while correcting for participant characteristics such as age, time poststroke or SCI, and stroke or SCI severity (FIM or SCIM). The same process will be used for the secondary and stroke-specific outcome measures. As secondary outcomes will be interpreted as exploratory, no formal multiplicity correction will be applied. Additionally, exploratory subgroup analyses will be considered based on pathology.

Descriptive statistics will be used to characterize usual care by group and over time based on the prospectively collected FITT-principles based therapy diaries, ensuring transparent reporting of the therapy that will be received during follow-up. In addition, descriptive statistics will be used to quantify, for the intervention group, the proportion of the 90-hour program corresponding to TOT.

The Leuven Biostatistics and Statistical Bioinformatics Center provided statistical support during this trial’s design and provides ongoing statistical support during this trial.

### Health Economic Evaluation

To evaluate the cost-effectiveness of the INTeRAcT program, a trial-based cost-utility analysis will compare the costs and effects of the intervention and control groups over a 9-month time horizon postbaseline. Additionally, a Markov model will be used to estimate cost-effectiveness over a lifetime horizon.

The health economic data, including costs and effects, will be collected using a combination of methods. Baseline data will be gathered at T0. The cost data will be collected during the 9-month follow-up period through self-completion questionnaires and diaries. The diaries will be completed during the first and the ninth period of this study, while a digital questionnaire will be sent via mail monthly during the intervening months. Resource use related to the intervention will be obtained directly from the rehabilitation center. The effects will be measured at 3 assessment time points: T0, T1, and T2, with additional data digitally surveyed each month during the 9 follow-up periods (Figures 1-3). To reduce missing or erroneous data, the questionnaires and diaries are reviewed monthly. In case of missing or unclear information, the participant will be contacted for clarification.

#### Data: Costs and Utilities

This evaluation will incorporate costs and effects during this trial and follow-up period, adopting both health care and societal perspectives. Both perspectives will include costs covering expenses borne by health insurance as well as patient copayments. The health care perspective will include all direct costs, both medical and nonmedical, while the societal perspective will encompass all costs, including direct medical and nonmedical costs as well as indirect costs.

The direct medical costs include all costs for treatment and follow-up from the health care perspective, and all out-of-pocket contributions by the participant. The following types of resource use will be collected: primary care visits, emergency visits, inpatient stays, outpatient services, medication, diagnostic tests, and medical or ADL aids.

Direct nonmedical costs include transportation and accommodation expenses, as well as home care assistance.

Indirect costs comprise productivity loss (eg, number of days away from work) and productivity losses due to informal caregiving. The productivity losses of the participants and informal caregivers will be valued using the human capital approach [134]

The effects will be expressed in Quality-Adjusted Life Years (QALYs). To calculate QALYs, health-related quality of life (QoL) scores are multiplied by the length of time spent in a particular health state. For example, 1 QALY represents 1 year of life in perfect health. Health-related QoL will be collected through the EQ-5D-5L questionnaire [113] and quantified in utilities derived from the national values of the EQ-5D-5L [135]. QALYs will be calculated using the area under the curve method.

To estimate the costs corresponding to the participants’ resource use, participants will be asked to provide invoices related to these expenses to ensure accurate cost reporting. Additionally, national tariffs will be used for valuation (NHIDI, BCFI, National feedback reports on hospitalizations, national cost per working hour, national cost for transportation, etc).

#### Data Analysis

First, a trial-based health economic evaluation will be conducted to assess the cost-effectiveness of the intensive, personalized rehabilitation program compared to usual care. Individual participant data from both the intervention and control group will be used to estimate the costs and health effects of the intensive personalized rehabilitation program compared to usual care from baseline up to 9 months of follow-up. The cost-effectiveness of the intervention will be expressed in incremental cost per QALY gained, calculated as the incremental cost-effectiveness ratio (ICER):


$$ICER=\frac{\left({\text{mean expected cost}}_{\text{intervention}}-{\text{mean expected cost}}_{\text{control}}\right)}{\left({\text{mean expected effect}}_{\text{intervention}}-{\text{mean expected effect}}_{\text{control}}\right)}=\frac{€}{\text{QALY}}$$

The robustness of the results will be evaluated through a deterministic and probabilistic sensitivity analysis of both costs and effects. A univariate deterministic sensitivity analysis will be conducted to assess the relative impact of individual cost components on the ICER. The results will be visually presented using tornado diagrams. A probabilistic sensitivity analysis will be performed using bootstrapping with replacement, with a minimum of 1000 iterations, to estimate the 2.5% and 97.5% percentiles of the ICER distribution. As all costs occur within a 1-year timeframe, no discounting will be applied. All bootstrapped ICERs will be visualized on a cost-effectiveness plane to assess the uncertainty surrounding the ICER and the probability that the intervention is cost-effective at various willingness-to-pay thresholds. To illustrate these probabilities of acceptable ICERs, a cost-effectiveness acceptability curve will be used. In addition, exploratory subgroup analyses will be considered based on pathology, type and severity of stroke and SCI, and total rehabilitation dose.

Given the extensive data collection in this trial, some missing data may occur; therefore, appropriate data imputation techniques will be applied [136]. The analysis will be conducted using R (R Foundation) software, following the Belgian guidelines for health economic evaluations [137], and will be reported according to the Consolidated Health Economic Evaluation Reporting Standards [138].

Second, we will perform a model-based health economic evaluation. Conventional practice guidelines for cost-effectiveness analysis recommend using a time horizon that is sufficiently long to capture all relevant costs and outcomes, particularly for interventions targeting chronic conditions. In this case, a lifetime horizon as the base case analysis is often suggested [139,140]. As the absence of long-term follow-up data should not justify the failure to extend the time horizon to a relevant duration [140], a Markov model will be developed. This approach will allow us to account for the expected costs and health outcomes in both intervention and control groups beyond the follow-up period, to estimate the cost-effectiveness over a lifetime horizon. Estimates of resource use and utilities for each health state are derived from international peer-reviewed literature. Special attention will be given to the transferability of this data to the Flemish health care context, validated through monitoring within the research consortium. Discount rates of 3% for costs and 1.5% for utilities will be applied, following the Belgian guidelines [137]. In line with the trial-based evaluation, probabilistic sensitivity analyses will be conducted to account for uncertainty around the input parameters. R software to develop the model, calculate the incremental cost per QALY, and perform the probability sensitivity analysis.

Lastly, several scenario analyses will be conducted to simulate various implementation models of the intensive program compared to current usual care, such as providing 1‐2 intensive rehabilitation boosts per year, rather than dispersing the same therapy volume across an entire year (eg, 1‐2 h of therapy each week for a year).

A budget-impact analysis will also be performed to assess how the introduction of this intensive therapy might affect the care budgets. This analysis will consider (1) the annual incidence of the specific population for whom this therapy is suitable, (2) the number of centers required to provide this therapy, (3) the planning timescale, and (4) the total cost of expanding this therapy across Flanders.

### Process Evaluation

The main objective of the process evaluation is to discover how complex interventions, such as INTeRAcT, function in practice and how they can be effectively transferred into clinical practice and policy when proven successful. This process evaluation will be guided by the Medical Research Council framework [88,90]. To gain insight into the implementation process of INTeRAcT, a mixed-methods approach will be adopted, incorporating both qualitative and quantitative methods. Data collection takes place during and for 4 weeks after the intervention (Figures 1-3).

The goals of the process evaluation are (1) to explore the quality and fidelity of the implementation by examining what is delivered and how it is delivered; (2) to understand whether, how, and why the therapy has an impact, by exploring both participants’ and health care providers’ perspectives on the program; (3) to analyze the context and the interaction between the program and the real-world context by identifying facilitators and barriers to the implementation and sustainability of the program, including recommendations for future implementation; and (4) to assess the transferability of results by examining participants’ and therapists’ perceived experiences and impact of the intervention. The latter goal will be supported by a quantitative analysis of the data and a full health economic evaluation from a societal perspective, aiming at informing reimbursement decisions for the therapy (see previous section Health Economic Evaluation).

#### Data: Structured Diaries, Semistructured Interviews, and Observations

Structured therapy diaries will provide information on how the intervention is delivered. These diaries, completed by the treating therapists, will capture data on the content of therapy, including the number and type of exercises given, the duration of therapy sessions, and other relevant treatment characteristics.

Semistructured interviews will be conducted to assess the quality of the therapy as experienced by both patients and therapists. Furthermore, these interviews will explore the perceived benefit and relevance of the intervention, elicit suggestions for its implementation in routine clinical practice, assess satisfaction and acceptability by identifying facilitators and barriers, and explore stakeholders’ expectations regarding the sustainability of the program. Therapists will also be invited to reflect on any adaptations made to the rehabilitation program.

Observations will be used to gain a deeper understanding of the content and delivery of the intervention. Further, 14 participants will be observed during therapy sessions by an unblinded researcher using a structured observation scheme. Each type of therapy block, upper and lower limb therapy (with and without technology), cardiovascular fitness training, goal-oriented training, and self-management, will be observed once per week over the 3-week intervention period. This will result in approximately 6 to 7 hours of observation per participant per week. These observations will also support the identification and interpretation of variations in outcomes across different settings and contexts.

The structured diaries, semistructured interview guides, and observation schemes were developed by the research team based on the literature [92,141-143] and expert opinions, and were piloted before data collection.

#### Data Analysis

Descriptive statistics will be used to report the quantitative data, and analyses will be performed using IBM SPSS Statistics for Windows. Qualitative data will be analyzed using both content analysis, a deductive analysis based on the defined indicators, and thematic analysis, an inductive analysis where themes will be generated from the data by the researchers using a generic qualitative approach. The aim is to interview approximately 16 participants and the 4 treating therapists to achieve meaning saturation [144], although the final sample size will be determined by saturation. Interviews will preferably be conducted face-to-face, depending on the availability of the interviewees; online interviews may also be arranged. All interviews will be audio-recorded, transcribed verbatim, and analyzed using NVivo (Lumivero).

To gain a more in-depth understanding of the implementation process, different types of data triangulation will be used: (1) methodological triangulation, using a combination of interviews and observations; (2) data triangulation, incorporating perspectives from both participants and therapists; (3) theoretical triangulation, involving researchers from different disciplines to interpret the data from varied perspectives; and (4) investigator triangulation, engaging multiple researchers in the data analysis process. Throughout the research, findings will be regularly discussed within the research team.

### Ethical Considerations

#### Ethical Approval

This study obtained ethical approval from the Ethics Committee Research of KU/University Hospitals Leuven, Belgium (registration number: B3222021000614, internal reference: S67164), as well as from the local ethics committee of the site, AZ Herentals, on June 13, 2023. This study will be conducted in accordance with the Declaration of Helsinki. The currently approved protocol is version 5, dated March 15, 2024. All participants provide written informed consent before enrollment (Multimedia Appendix 1). No financial compensation was provided aside from 3 weeks of free high-dose therapy and reimbursement of expenses (eg, transport costs).

Throughout the clinical trial, an annual progress report (or additional reports upon request) will be submitted to the ethics committee, providing an overview of all (serious) AEs that occurred during the reporting period, along with newly available safety information. This study will adhere to the approved protocol, current International Council for Harmonisation and International Council for Harmonisation–Good Clinical Practice guidelines, applicable regulatory requirements, the European General Data Protection Regulation 2016/679, and relevant Belgian laws implementing the General Data Protection Regulation. The currently approved protocol is version 5, dated March 15, 2024. Protocol amendments will be submitted to all involved ethical committees. This protocol was developed following the SPIRIT (Standard Protocol Items: Recommendations for Interventional Trials) 2013 checklist (Checklist 2).

#### Data Handling and Dissemination

Data collection, handling, processing, and transfer will be performed in compliance with applicable regulations, clinical study guidelines, and internal procedures. The details of data handling and data flow management are outlined in this study-specific data management plan. Data will be pseudonymized, and (electronic) case report forms shall under no circumstances capture personal identifiers. Paper documents will be stored in a locked cupboard.

The principal investigator (PI), GV, is responsible for registering this study. In addition, the PI will fulfill its ethical obligation to disseminate and make the research results publicly available. As such, the PI is accountable for the timeliness, completeness, and accuracy of the reports. A variety of means will be used to disseminate the results, including academic publications, conference presentations, written reports, and communication at various stakeholder events. The full pseudonymized dataset will become available in a restricted-access repository. The dataset could be made available for other research after submitting a written request to the PI, approval of an ethical committee, and based on a data transfer agreement. The first paper will be based on the combined outcomes of the stroke and SCI populations, covering both clinical and health economic aspects (trial- and model-based). Subsequently, publications will follow focusing on stroke-related clinical outcomes, the budget impact analysis, and the process evaluation.

## Results

The funding of the project started in February 2023. The project agreement ended in September 2025, while the personal fellowship continues until October 2027. Recruitment took place between June 2023 and September 25, 2024, enrolling 102 participants. The last clinical visit occurred on July 9, 2025. Submission of this paper occurred after completion of recruitment because the process evaluation methodology was integrated later in the protocol, within the planned timeline of the fellowship. No data analyses were conducted before finalizing this protocol. Data analysis is ongoing.

## Discussion

This study investigates whether the INTeRAcT motor program is effective in improving functional independence in daily living, cost-effective, and suitable for implementation in other rehabilitation centers, compared to usual care, in individuals with chronic stroke or SCI.

This study protocol was created in close collaboration with clinical practitioners, scientific researchers, patients, and policymakers. One of the key stakeholders is the National Institute for Health and Disability Insurance, Belgium’s federal agency responsible for health and disability insurance, ensuring a direct link with health policy and reimbursement discussions. Different core elements of evidence-based research were integrated in the development of this study protocol [145]. The initial inspiration for the study arose from clinical practice and was further shaped by building upon previous work by Ward et al [24,92]. The protocol was refined through the incorporation of therapists’ experiences and feedback from patients.

A second strength of this project is its alignment with the phases outlined in the Medical Research Council framework [90]. This study builds on prior research and contributes to the next phase of intervention development by evaluating its clinical effectiveness, cost-effectiveness, and implementation through a structured process evaluation [88]. The broad assessment across various ICF domains ensures a holistic understanding of participants’ motor function, ADLs, and societal participation. In addition, the process evaluation adds value to the literature, as even promising outcomes may face challenges when translated into different rehabilitation contexts. Finally, the inclusion of a health economic evaluation from both societal and health care perspectives will generate robust evidence on the cost-effectiveness of the INTeRAcT program, supporting policymakers in making informed decisions regarding its broader implementation.

A third strength is the program’s strong patient-centered approach, with therapy blocks built around individual goals and guided by a clear, structured methodology. Each therapy week offers a balanced combination of the core components: upper-limb therapy with and without advanced technology (5 hours each), lower-limb therapy with and without technology (5 hours each), goal-oriented functional training (5 hours), cardiovascular fitness training (2.5 hours), and self-management education (2.5 hours). This consistent yet adaptable structure ensures both standardization and individualization based on personal goals. Group-based sessions offer opportunities for peer interaction and shared learning without compromising individual needs. Furthermore, the broad geographical inclusion of participants across Belgium, supported by tailored transportation solutions, enhances the generalizability of the results and promotes equitable access.

Lastly, by evaluating the full rehabilitation program rather than isolated components, this study adopts a whole-systems approach that more accurately reflects clinical reality. This enhances external validity and supports the integration of findings into daily practice, should the intervention prove effective.

On the other hand, certain limitations must be acknowledged. First, the intervention is delivered in a single clinical center within the context of this study, which may limit the generalizability of the results and the replicability of the INTeRAcT program in other settings. To mitigate this, a process evaluation is incorporated into the study to explore mechanisms of change, relevant contextual factors, and identify potential facilitators and barriers to implementation of the program in different clinical environments [91].

Second, this study includes 2 patient populations, resulting in a heterogeneous sample. However, both populations share the common characteristic of having an acquired neurological condition in adulthood, typically presenting with similar primary symptoms such as paresis, motor and sensory impairments, and secondary complications including spasticity and cardiovascular issues. Together, these symptoms significantly affect patients’ mobility and functional independence, leading to comparable rehabilitation needs. Moreover, the recovery trajectories in both groups are comparable, with a critical window for motor recovery typically occurring in the first months after onset [14,15,18]. Nevertheless, evidence indicates that motor improvements remain achievable in the chronic phase, driven by exercise-dependent plasticity [19,23,24,34]. The INTeRAcT program also incorporates principles of motor learning, which are relevant and applicable to both patient populations [14,146]. This broader inclusion enhances the generalizability of the findings to other neurological conditions, thereby increasing the relevance and applicability of the results beyond the specific populations studied. Therefore, evaluating both populations within a single trial can be considered appropriate.

Third, as this study is designed as a pragmatic trial, the control group receives usual care, reflecting the current real-life clinical context. The inherent variability in usual care may influence clinical outcomes; therefore, both the total amount of therapy and the content delivered are documented, as outlined in the Methods section.

Lastly, although the goal-directed nature of the program provides a clear source of motivation for patients [147,148], the self-enrollment procedure may attract more motivated individuals, potentially introducing selection bias. However, this phenomenon likely reflects clinical reality, where patients with higher intrinsic motivation are more inclined to participate in continued care. The process evaluation will provide crucial insights into the extent to which patient motivation contributes to the observed outcomes.

Through this study, we aim to improve rehabilitation in the chronic phase of stroke and SCI. Our goals are to deliver robust evidence to support reimbursement of this motor rehabilitation package and to facilitate its implementation in other rehabilitation centers. We hope that our findings will promote an increased rehabilitation dose and intensity, the tailoring of interventions to individual patient needs, and the integration of rehabilitation technologies in the chronic phase, both in Belgium and beyond.

## Supplementary material

10.2196/93234 Multimedia Appendix 1 Informed consent participants.

10.2196/93234 Multimedia Appendix 2 Overview of demographic, socioeconomic, and pathology-specific variables.

10.2196/93234 Checklist 1 TIDieR checklist INTeRAcT intervention.

10.2196/93234 Checklist 2 SPIRIT checklist.

10.2196/93234 Peer Review Report 1 Peer review report by the College of Medical Directors, the National Institute for Health and Disability Insurance (NIHDI/RIZIV, Belgium).

## Abbreviations

ADL: activity of daily living
AE: adverse event
COPM: Canadian Occupational Performance Measure
FIM: Functional Independence Measure
FITT: Frequency, Intensity, Time, and Type
GAS: Goal Attainment Scaling
ICER: incremental cost-effectiveness ratio
ICF: International Classification of Functioning, Disability, and Health
INTeRAcT: Intensive Rehabilitation Programme Integrating Advanced Technology
MD: medical doctor
PI: principal investigator
QALY: Quality-Adjusted Life Year
SCI: spinal cord injury
SCIM: Spinal Cord Independence Measure
SMART: Specific, Measurable, Achievable, Relevant, and Time-Bound
SPIRIT: Standard Protocol Items: Recommendations for Interventional Trials
T0, T1 and T2: Measurement Timepoints: baseline, after 3 weeks (postintervention), after 9-months (follow‑Up)
TIDieR: Template for Intervention Description and Replication
TOT: time on task
