# Supplementary material for: Impact of a Personalized, High-Dose, Intensive Motor Rehabilitation Program, Integrating Advanced Technology for Adults With Central Neurological Conditions (INTeRAcT): Protocol for a Single-Blind Randomized Controlled Trial With a Clinical, Health Economic, and Process Evaluation
Source: JMIR Res Protoc. 2026 May 4;15:e93234. doi: 10.2196/93234 (PMC13151458; doi:10.2196/93234)
Supplement: Multimedia Appendix 1 — Informed consent participants. [file resprot-v15-e93234-s001.pdf]

DEPARTEMENT REVALIDATIEWETENSCHAPPEN
TERVUURSEVEST 101 BUS 1501, 3001 HEVERLEE

Informatie- en toestemmingsformulier

Effect van een intensief revalidatieprogramma met integratie van technologie voor volwassenen met een centraal neurologische aandoening

Onderzoekers: Prof. Dr. Geert Verheyden
Prof. Dr. Koen Putman & Prof. Dr. Maaike Fobelets

Opdrachtgever: KU Leuven, Faculteit Bewegings- en Revalidatiewetenschappen
Departement Revalidatiewetenschappen
Onderzoeksgroep Neurorevalidatie
Tervuursevest 101, bus 1501
3001 Leuven

Ethische Commissie Onderzoek UZ/KU Leuven

Titel van de studie: **Effect van een intensief revalidatieprogramma met integratie van technologie voor volwassenen met een centraal neurologische aandoening:**
klinische, gezondheidseconomische en procesevaluatie

Opdrachtgever: *KU Leuven, Biomedische Wetenschappen Departement Revalidatiewetenschappen*

Onderzoeksinstellingen*: KU Leuven, AZ Herentals en Vrije Universiteit Brussel (VUB)*

Comité voor Medische Ethiek: *Ethische Commissie onderzoek UZ / KU Leuven*

Lokale therapeuten-onderzoekers:

KU Leuven:
Hoofdonderzoeker: *Prof. Geert Verheyden*
Trial manager: Marjan Coremans
Wetenschappelijk medewerker: Laura Pattyn

AZ Herentals:
Onderzoeker: Dr. Ingue Allewijn
Therapeut: Agaat
Therapeut: Femke
Therapeut: Floris
Trial therapist (KUL): Laure

VUB:
Prof. Dr. Koen Putman
Prof. Dr. Maaike Fobelets

I. Noodzakelijke informatie voor uw beslissing om deel te nemen

Inleiding

U wordt uitgenodigd om deel te nemen aan een wetenschappelijk studie. Deze klinische studie wordt georganiseerd om te onderzoeken of intensieve revalidatie (zo'n 90 uur verspreid over 3 weken) met integratie van technologie, zinvol is in een chronische fase na een centraal neurologische aandoening. Deze studie wordt mede gefinancierd door het Rijksinstituut voor ziekte- en invaliditeitsverzekering (RIZIV).

Revalidatie na een centraal neurologische aandoening is zinvol en noodzakelijk, op elk moment na de aandoening. Revalidatie brengt de patiënt tot een bepaald niveau, maar voor veel gehospitaliseerde patiënten die daarna opgenomen worden in een revalidatieprogramma, is dit niet het niveau van voor de aandoening. Er blijven dus beperkingen als patiënten uit het revalidatiecentrum ontslagen worden naar de thuissituatie (chronische fase: > 6 maand). Hoewel ze recht hebben op ambulante therapie in deze chronische fase, zal dit therapie zijn aan een veel lagere frequentie. Bovendien verliezen patiënten in een chronische fase, op lange termijn, functionaliteit en evolueren zo naar een lager niveau van activiteit. Intensievere revalidatie in een chronische fase is dus aangewezen. Een verbetering van functionaliteit zal worden bereikt door intensief te oefenen dankzij een toename in onder meer kracht, uithouding, flexibiliteit en coördinatie. Algemeen vermoeden we hoe intensiever kan gewerkt worden, hoe meer verbetering gezien kan worden. Wij bieden daarom een revalidatiepakket aan waarbij 90 uur therapie gegeven wordt, gespreid over 3 weken. De revalidatie zal een combinatie zijn van standaard therapie en hoog technologische toestellen (staprobot, armrobot…) . Het gebruik van hoog technologische toestellen levert oefening aan hoge intensiteit, maar hiernaast is ook standaard therapie noodzakelijk om de overgang te maken naar dagelijkse activiteiten.

Voordat u beslist over uw deelname aan deze studie willen we u wat meer informatie geven over wat dit betekent op organisatorisch vlak en wat de eventuele voordelen en risico's voor u zijn. Zo kan u een beslissing nemen op basis van de juiste informatie. Dit wordt "geïnformeerde toestemming" genoemd.

Wij vragen u de volgende pagina's met informatie aandachtig te lezen. Hebt u vragen, dan kan u terecht bij de therapeut-onderzoeker. Dit document bestaat uit 3 delen: essentiële informatie die u nodig heeft voor het nemen van uw beslissing, uw schriftelijke toestemming en bijlagen waarin u meer details terugvindt over bepaalde onderdelen van de basisinformatie.

Als u aan deze studie deelneemt, moet u weten dat:

- Deze klinische studie opgesteld is na evaluatie door meerdere ethische comités.
- Uw deelname vrijwillig is; er kan op geen enkele manier sprake zijn van dwang. Voor deelname is uw ondertekende toestemming nodig. Ook nadat u hebt getekend, kan u de therapeut-onderzoeker laten weten dat u uw deelname wilt stopzetten. De beslissing om al dan niet (verder) deel te nemen zal geen enkele negatieve invloed hebben op de kwaliteit van de zorgen noch op de relatie met de behandelende therapeut of arts.
- De gegevens die in het kader van uw deelname worden verzameld, zijn vertrouwelijk. Bij de publicatie van de resultaten zal de bescherming van uw identiteit worden verzekerd
- Er worden u geen kosten aangerekend voor specifieke behandelingen, bezoeken / consultaties, onderzoeken in het kader van deze studie.
- Er een verzekering afgesloten is voor het geval dat u schade zou oplopen in het kader van uw deelname aan deze klinische studie.
- Indien u extra informatie wenst, u altijd contact kan opnemen met de therapeut-onderzoeker of een medewerker van zijn of haar team.

Aanvullende informatie over uw “Rechten als deelnemer aan een klinische studie” vindt u in bijlage 1.

Doelstellingen en beschrijving van het studieprotocol

Wij nodigen u uit om deel te nemen aan een klinische studie waarin de voordelen van een nieuw revalidatiepakket voor patiënten in de chronische fase na een beroerte of dwarslaesie wordt onderzocht. Aan deze klinische studie zullen 100 mensen met een beroerte of dwarslaesie deelnemen in België.

Het doel van deze studie is om de klinische effecten (vb. functionaliteit in het dagelijkse leven, vooruitgang in persoonlijke doelen, levenskwaliteit…) na te gaan van een nieuwe gepersonaliseerd intensief revalidatieprogramma. Daarnaast zal ook de kosteneffectiviteit van het programma beoordeeld worden. Ten slotte zal er een procesevaluatie uitgevoerd worden. Binnen een procesevaluatie wordt gekeken hoe vlot het invoeren van de intensieve revalidatie in het revalidatiecentrum verloopt en waar er zich nog problemen voordoen.

Tijdens het verloop van de studie mag u de door uw arts voorgeschreven kinesitherapeutische behandelingen voortzetten. Ook na afloop van de studie kan u eventueel voorgeschreven therapie verderzetten.

Indien u in de interventie groep terecht komt zullen we u vragen om gedurende 3 weken, 5 dagen per week naar AZ Herentals te komen om het intensief revalidatie programma te volgen (standaard therapie + technologie) en vervolgens gedurende 8 maanden, maandelijks te worden opgevolgd (digitaal contact). Start u in de controle groep wordt u eerst 9 maanden opgevolgd en kan u nadien de 3 weken intensieve revalidatie ontvangen. Bij beide groepen wordt bij het begin van de studie, na 3 weken en na de follow-up periode van 9 maanden de functionaliteit in het dagelijkse leven, mobiliteit, functie van bovenste en/of onderste ledematen onderzocht.

Om aan deze studie te kunnen deelnemen, moet u voldoen aan de volgende criteria:

- Een diagnose van een centrale neurologische aandoening, gesteld door een specialist. De aandoening kan een beroerte (CVA) of dwarslaesie (DWL) omvatten;
- Ten minste 3 maanden na ontslag uit het ziekenhuis/revalidatiecentrum, thuiswonend
- Een herstel van maximaal 85% van de onafhankelijkheid in het dagelijks functioneren (FIM/SCIM);
- Een normale functionele toestand voorafgaand aan de pathologie (Barthel Index > 85/100);
- Unilaterale of bilaterale zwakte van de aangetaste bovenste of onderste ledematen (geen maximumscore in alle aangetaste ledematen = Motricity Index: score 5 op 3/3 segmenten)
- De klinische mogelijkheid hebben om oefentherapie te volgen met de beschikbare technologie, beoordeeld door revalidatiearts;
- Leeftijd van > 18 jaar;
- Voldoende cognitief vermogen om revalidatieoefeningen/games te begrijpen en geïnformeerde toestemming te geven, beoordeeld door revalidatiearts;
- Geen andere neurologische of musculoskeletale aandoeningen die het protocol kunnen beïnvloeden, beoordeeld door revalidatiearts;
- Vermogen om bewegingen tegen de zwaartekracht in te initiëren in de bovenste of onderste ledematen;
- Geen ernstige visuele beperking, communicatie-, cognitieve en taalvormingsproblemen die het interventie- en meetproces zouden verhinderen, beoordeeld door revalidatiearts;
- Geen aandoening die naar de mening van de onderzoeker de veiligheid van de deelnemer of de naleving van het studieprotocol in gevaar kan brengen, beoordeeld door revalidatiearts.

Er zullen een aantal testen uitgevoerd worden om zeker te zijn dat u aan alle criteria voldoet.

Verloop van de studie (in AZ Herentals)

Uw deelname aan de studie zal 3 volledige weken in beslag nemen, met daarna een opvolg periode tot 9 maanden na start van de (controle) interventie. In onderstaande figuur kan u het verloop van de studie zien.

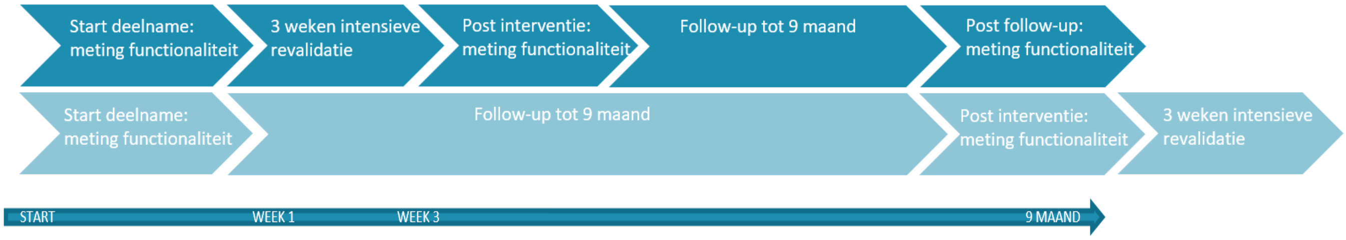

Verloop studie: donker blauw: Interventie; licht blauw: Controle groep

Indien u besluit deel te nemen aan de studie en aan alle voorwaarden voor deelname voldoet, zal u onderstaande testen en onderzoeken doorlopen:

a) Randomisatie

Bij de start van de studie worden de studiedeelnemers willekeurig in 2 verschillende groepen verdeeld: een controle groep en een interventie groep. De interventiegroep start met 3 weken intensieve therapie en wordt daarna opgevolgd tot 9 maanden. De controle groep mag zijn standaard therapie verderzetten gedurende 9 maanden. Na de afloop van de studie krijgen zij ook de mogelijkheid om deze intensieve revalidatie van 3 weken te volgen. Er is geen mogelijkheid om te kiezen en op voorhand te weten in welke groep u zal belanden. Na het eerste meetmoment krijgt komt u te weten in welke groep u zal belanden.

b) Metingen en vragenlijsten

Bij de start van uw deelname aan de studie zullen enkele persoonlijke, ziekte-gerelateerde en gezondheidseconomische kenmerken verzameld worden, zoals uw leeftijd, geslacht, tijdsperiode sinds uw beroerte/dwarslaesie, handdominantie, opleiding, werk…

Voor de start van de therapie, zullen we uw functionaliteit en onafhankelijkheid in het dagelijkse leven bevragen. Daarnaast zullen we u vragen om een vragenlijst in te vullen die peilt naar uw kwaliteit van leven en wordt er gekeken naar uw mobiliteit en functie van bovenste en/of onderste ledematen. Alle testen en vragenlijsten worden de samen met de onderzoeker overlopen en ingevuld op papier. Verder zal u ook gevraagd worden om zelf doelen op te stellen dat u wil bereiken tijdens de revalidatie.

Het verzamelen van deze gegevens, het invullen van de vragenlijsten en het afnemen van de testen zal in totaal ongeveer 2 uur in beslag nemen. Afhankelijk van de praktische haalbaarheid kunnen de metingen plaatsvinden in AZ Herentals, KU Leuven (Tervuursevest 101, Leuven) of bij de deelnemer thuis.

In het schema op pagina 6 – 7 kan u een samenvatting vinden van de testen en vragenlijsten die afgenomen zullen worden.

	Testen		Inhoud	Duur
**Klinische data CVA + DWL**	Functional Independence Measure (FIM)	Functionele mogelijkheden: observatie therapeut	Meet de onafhankelijkheid in het dagelijkse functioneren na beroerte.	10-20 min
	Spinal Cord Independence Measure (SCIM)	Functionele mogelijkheden: observatie therapeut	Meet de onafhankelijkheid in het dagelijkse functioneren na dwarslaesie.	10-20 min
	Canadian Occupational Performance Measure (COPM)	Functionele mogelijkheden: perceptie deelnemer	Deze vragenlijst meet de inschatting van het eigen functioneringsvermogen in het dagelijkse leven doorheen de tijd.	20-30 min
	De Goal Attainment Scaling (GAS)	Persoonlijke revalidatie doelen	Het is een geïndividualiseerde evaluatiemethode, waarmee het individuele behandeldoel van een persoon wordt vastgelegd en na afloop gescoord op het bereiken van dat behandeldoel.	20-30 min
	EQ-5D-5L	Kwaliteit van leven	De EQ-5D is een gestandaardiseerd instrument waarmee op vijf gezondheidsniveaus een score wordt gegeven. Verder moet de persoon aangeven hoe hij zijn gezondheidstoestand op een schaal van 0 tot 100 ervaart.	5-10 min
	Fatigue severity scale (FSS)	Vermoeidheid	Deze meetschaal wordt gebruikt om de ervaren ernst van de vermoeidheid-symptomen in de afgelopen week in verschillende dagelijkse situaties na te gaan.	5-10 min
**Klinische data CVA**	6-Minuten Wandel Test (6MWT)	Cardiovasculaire fitness	De 6 minuten wandeltest wordt gebruikt om de functionele capaciteit te meten: de max. afstand iemand kan afleggen in 6min.	10 min

	Action research arm test (ARAT)	Functionele mogelijkheden bovenste lidmaat	Test voor de arm-/handvaardigheid: verschillende voorwerpen grijpen en verplaatsen met verschillende handgrepen.	10-15 min
	Fugl-Meyer motor assessment (FMA)	Functionele mogelijkheden bovenste & onderste lidmaat	Een performance test om de mate van beperking in functies te bepalen van bovenste en onderste lidmaat.	30 min
	Functional Ambulation Classification (FAC)	Mobiliteit/bewegen	Met deze schaal wordt de mate van zelfstandigheid van lopen van de persoon geëvalueerd (met/zonder loophulpmiddel).	5 min
	10 Meter Wandel Test (10MWT)	Mobiliteit/bewegen	Bij de 10 meter looptest wordt de snelheid van het comfortabel lopen en de maximale loopsnelheid gemeten over een afstand van 10 meter.	5 min.
	Stroke self-efficacy questionnaire (SSEQ)	zelfredzaamheid	Evalueer het vertrouwen van individuen in het uitvoeren van activiteiten van het dagelijks leven.	10-15min.
**Gezondheidseconomische data**	Gezondheidseconomische vragenlijst/dagboek (GEV/D)	Gezondheidseconomische gegevens	Aan de hand van deze gegevens willen we de kosten en effecten van de therapie in kaart brengen tijdens de interventie en follow-up periode.	5-10 min
**Therapie dagboek**	Therapie dagboek	Frequentie<br>Duur<br>Content	Aan de hand van dit dagboekje wordt de frequentie, duur en content van de verkregen therapie tijdens de interventie en follow-up periode.	2-5 min

c) Therapie & follow-up

Interventie groep:

Vervolgens krijgt u 3 weken intensieve revalidatie; dit is een combinatie van standaard therapie en hoog technologische toestellen. De toestellen voldoen aan alle nodige vereisten, zoals een CE markering. Het zijn erkende apparaten. Een ruime waaier aan toestellen zal worden gebruikt tijdens de therapie, onder andere fitnessapparatuur, loopband, staprobots, arm-/handrobot… De therapeuten zijn opgeleid om de revalidatie te geven, alsook met de toestellen te werken.

De training duurt 6 uur per weekdag en zal telkens op dezelfde manier worden opgebouwd zoals te zien op het schema hieronder. Tijdens de therapie wordt de therapiesessie mogelijks geobserveerd in kader van de procesevaluatie.

Na de 3 weken intensieve revalidatie, zal u nog tot 9 maand na de eerste meting worden opgevolgd. Tijdens deze maanden zal je ook je therapie dagboek invullen telkens je therapie hebt gevolgd, waarin de frequentie, tijdsduur en content van de therapie wordt beschreven. Niet tijdens de eerste 3 weken intensieve therapie, dan vult de therapeut van de studie dit voor jou in.

Daarnaast zal u een dagboek en vragenlijst ontvangen over uw gezondheidseconomische gegevens (vb. hoe vaak bent u de afgelopen maand op consultatie geweest bij uw huisarts?). Gedurende de eerste en laatste maand zal u wekelijks het dagboekje moeten invullen. In de maanden daartussen zal u maandelijks een vragenlijst invullen (digitaal/telefonisch) over uw gezondheidseconomische gegevens en over uw kwaliteit van leven.

Tot slot zal u mogelijks gecontacteerd worden door een onderzoeker voor een interview over uw ervaring met de intensieve revalidatie. Dit interview zal plaatsvinden 2 – 6 weken na uw intensieve revalidatie.

	Maandag	Dinsdag	Woensdag	Donderdag	Vrijdag
09.30	Cardiovasculaire fitnesstraining				
10.00	Therapie bovenste lidmaat				
10.30					
11.00	Therapie bovenste lidmaat + technologie				
11.30					
12.00	Middageten				
12.30					
13.00	Therapie onderste lidmaat				
13.30					
14.00	Therapie onderste lidmaat + technologie				
14.30					
15.00	Individuele training volgens doelstellingen				
15.30					
16.00	Zelf-management				

<table>
<tr><th rowspan="2">Maand</th><th colspan="4">1</th><th rowspan="2">2</th><th rowspan="2">3</th><th rowspan="2">4</th><th rowspan="2">5</th><th rowspan="2">6</th><th rowspan="2">7</th><th rowspan="2">8</th><th rowspan="2">9</th></tr>
<tr><th>Week 1</th><th>Week 2</th><th>Week 3</th><th>Week 4</th></tr>
<tr><td>Therapie dagboek (bij contact)</td><td></td><td></td><td></td><td>x</td><td>x</td><td>x</td><td>x</td><td>x</td><td>x</td><td>x</td><td>x</td><td>x</td></tr>
<tr><td>Gezondheidseconomische dagboek (bij contact)</td><td colspan="4">x</td><td></td><td></td><td></td><td></td><td></td><td></td><td></td><td>x</td></tr>
<tr><td>Gezondheidseconomische + kwaliteit van leven vragenlijst (maandelijks)</td><td colspan="4"></td><td>x</td><td>x</td><td>x</td><td>x</td><td>x</td><td>x</td><td>x</td><td></td></tr>
</table>

X = Periode dat je de vragenlijst/dagboek moet invullen voor de ***interventie groep****.*

Controle groep:

Uw wordt gedurende 9 maand opgevolgd. U mag uw standaard therapie gewoon verderzetten.

Tijdens deze maanden zal je ook je therapie dagboek invullen telkens je therapie hebt gevolgd, waarin de frequentie, tijdsduur en content van de therapie wordt beschreven.
Daarnaast zal u een dagboek en vragenlijst ontvangen over uw gezondheidseconomische gegevens (vb. hoe vaak bent u de afgelopen maand op consultatie geweest bij uw huisarts?). Gedurende de eerste en laatste maand zal u wekelijks het dagboekje moeten invullen. In de maande daartussen zal u maandelijks een vragenlijst invullen (digitaal/telefonisch) over uw gezondheidseconomische gegevens en over uw kwaliteit van leven.

Maand	1	2	3	4	5	6	7	8	9
Therapie dagboek (bij contact)	x	x	x	x	x	x	x	x	x
Gezondheidseconomische dagboek (bij contact)	x								x
Gezondheidseconomische + kwaliteit van leven vragenlijst (maandelijks)		x	x	x	x	x	x	x	

X = Periode dat je de vragenlijst/dagboek moet invullen voor de ***controle groep****.*

<u>Risico's en ongemakken:</u>

Uw deelname aan deze studie houdt geen ernstige gezondheidsrisico's in. De training is vergelijkbaar met een kinesitherapeutische behandeling, maar intensiever: Daarom is mogelijk dat u tijdens of na de training onderstaande symptomen ondervind:

- Vermoeidheid, spierkrampen/toename van de spiertonus en pijn, zoals bij elke bewegingsoefening met een apparaat dat het lichaam en de spieren activeert. Dit soort symptomen zijn te verwachten en zouden moeten verdwijnen.
- Roodheid van de huid, lichte kneuzingen of kleine oppervlakkige drukletsels waar de apparatuur druk heeft uitgeoefend op de huid, door de harnassen of het gebruik van de revalidatierobots. Deze letsels worden gevolgd zoals in de standaardzorg: nauwlettend toezicht door de therapeut en gebruik van drukverbanden om ze te voorkomen.
- Orthostatische hypotensie: bloeddrukdaling door positieverandering (meestal door verticalisering: zitten/liggen naar staan).

- Veranderingen in blaas- en darmcontrole na activering van de deelnemer.

Voordelen:

Verder mag u niet verwachten dat uw deelname aan deze studie u persoonlijke voordelen zal opleveren, tenzij misschien een betere functionaliteit in het dagelijkse leven. Maar of de therapie zinvol is, wordt natuurlijk onderzocht en is nog niet bewezen in eerder onderzoek. Wel zal de informatie, die dankzij dit onderzoek verkregen wordt, bijdragen tot een beter begrip van gepersonaliseerde intensieve revalidatie in de chronische fase na de beroerte/dwarslaesie. Bovendien hopen wij op basis hiervan in de toekomst betere behandelingen te kunnen ontwikkelen.

Intrekking van uw toestemming

U neemt vrijwillig deel aan deze studie en u hebt het recht om uw toestemming voor gelijk welke reden in te trekken. U hoeft hiervoor geen reden op te geven. Wel kan het voor de therapeut-onderzoeker en de opdrachtgever nuttig zijn om te weten waarom u zich terugtrekt.

Het is ook mogelijk dat de therapeut-onderzoeker uw deelname aan de studie stopzet omdat hij/zij van mening is dat dit beter is voor uw gezondheid of omdat hij/zij vaststelt dat u zich niet aan de voorschriften voor deelname houdt.

Ook gebeurt het soms dat de bevoegde nationale of internationale autoriteiten, de ethische comités die aanvankelijk goedkeuring hadden gegeven voor de studie of de opdrachtgever de studie stopzetten omdat uit de verzamelde informatie blijkt dat de behandeling niet werkt of dat de onderzochte behandeling meer of ernstigere bijwerkingen veroorzaakt dan verwacht of voor een andere reden. Als u uw toestemming intrekt, zullen de gegevens bewaard blijven die tot op het ogenblik van uw stopzetting werden verzameld. Dit om de geldigheid van de studie te garanderen. Er zal geen enkel nieuw gegeven aan de opdrachtgever worden gegeven.

Als u aan deze studie deelneemt, vragen wij om:

- Ten volle mee te werken voor een correct verloop van de studie.
- Geen informatie over uw gezondheidstoestand, de geneesmiddelen die u gebruikt of de symptomen die u ervaart te verzwijgen.
- Uw therapeut-onderzoeker op de hoogte te brengen als men u voorstelt om aan een andere studie deel te nemen zodat u met hem/haar kan bespreken of u aan deze studie kunt deelnemen en of uw deelname aan de huidige klinische studie moet worden stopgezet.

Contact

Als u bijkomende informatie wenst, maar ook ingeval van **problemen** of als u **zich zorgen maakt.**	Hoofdonderzoeker:<br>*Geert Verheyden*<br>[…]<br>Coördinator van de studie:<br>*Marjan Coremans*<br>[…]
Voor praktische zaken tijdens **de 3 weken intensieve revalidatie** (vb. ziekte, te laat, afwezigheid…).<br><br>Verblijf, vervoer, verpleging…	Therapeuten (AZ Herentals):<br>[…]
Voor **studie gerelateerde zaken/vragen** voor of tijdens jouw deelname aan de studie: tijdens de intensieve revalidatie of gedurende de follow-up periode van 9 maanden.	Coördinator van de studie:<br>*Marjan Coremans*<br>[…]
In geval van **nood.**	Hoofdonderzoeker:<br>*Geert Verheyden*<br>[…]

Indien u vragen hebt over hoe wij uw gegevens gebruiken of uw recht op inzage, correctie, eventueel stopzetting van de verdere verwerking wil uitoefenen, dan kan u hiervoor steeds terecht bij de hoofdonderzoeker: Prof. Geert Verheyden.

Indien u naderhand nog bijzondere aandachtspunten heeft of klacht wenst neer te leggen, kan u terecht bij het privacy team van de KU Leuven op […].
Voor vragen met betrekking tot de rechten als deelnemer aan een studie, kunt u contact opnemen met de Ethische Commissie Onderzoek UZ/KU Leuven ([…]).

Een intensief revalidatieprogramma na een centraal neurologische aandoening

II. Geïnformeerde toestemming

Deelnemer

Ik verklaar dat ik geïnformeerd ben over de aard, het doel, de duur, de eventuele voordelen en risico's van de studie en dat ik weet wat van mij wordt verwacht. Ik heb kennis genomen van het informatiedocument en de bijlagen ervan.

Ik heb voldoende tijd gehad om na te denken en met een door mij gekozen persoon, zoals mijn huisarts of een familielid, te praten.

Ik heb alle vragen kunnen stellen die bij me opkwamen en ik heb een duidelijk antwoord gekregen op mijn vragen.

Ik begrijp dat mijn deelname aan deze studie vrijwillig is en dat ik vrij ben mijn deelname aan deze studie stop te zetten zonder dat dit mijn relatie schaadt met het therapeutisch team dat instaat voor mijn gezondheid.

Ik begrijp dat er tijdens mijn deelname aan deze studie gegevens over mij zullen worden verzameld en dat de therapeut-onderzoeker en de opdrachtgever de vertrouwelijkheid van deze gegevens verzekeren overeenkomstig de Europese en Belgische wetgeving ter zake.

Ik begrijp dat het uitvoeren van deze studie door KU Leuven het algemeen belang dient en de verwerking van mijn persoonsgegevens noodzakelijk is voor het uitvoeren van deze studie.

Ik geef toestemming dat er in het kader van deze klinische studie een geluidsopname wordt gemaakt van het interview om later door de onderzoeker uitgeschreven te worden. Deze data wordt volledig vertrouwelijk behandeld. Nadat het interview is uitgeschreven wordt de geluidsopname onmiddellijk verwijderd. Deze resultaten van deze gegevens zullen enkel in groep gerapporteerd worden en nooit individueel.

Ik begrijp dat mijn gegevens gedurende 25 jaar bewaard zullen worden.

Ik heb een exemplaar ontvangen van de informatie aan de deelnemer en de geïnformeerde toestemming.

Naam, voornaam, datum en handtekening van de deelnemer

____

Wettelijke vertegenwoordiger

Ik verklaar dat men mij heeft geïnformeerd over de vraag om een beslissing te nemen over deelname aan de klinische studie door de persoon die ik in diens beste belang vertegenwoordig, rekening houdend met zijn of haar mogelijke wens. Mijn toestemming is van toepassing op alle items opgenomen in het toestemmingsformulier voor de deelnemer.

Ik heb een exemplaar ontvangen van de informatie aan de deelnemer en de geïnformeerde toestemming.

Naam, voornaam en verwantschap met de vertegenwoordigde persoon:

Datum en handtekening van de wettelijke vertegenwoordiger

____

Therapeut-onderzoeker

Ik ondergetekende ______________________________________ therapeut-onderzoeker / bevoegde onderzoeksmederwerker, verklaar de benodigde informatie inzake deze studie mondeling te hebben verstrekt evenals een exemplaar van het informatiedocument aan de deelnemer te hebben verstrekt.

Ik bevestig dat geen enkele druk op de deelnemer is uitgeoefend om hem/haar te doen toestemmen tot deelname aan de studie en ik ben bereid om op alle eventuele bijkomende vragen te antwoorden.

Ik bevestig dat ik werk in overeenstemming met de ethische beginselen zoals vermeld in de laatste versie van de "Verklaring van Helsinki", de "Goede klinische praktijk" en de Belgische wet van 7 mei 2004 inzake experimenten op de menselijke persoon.

Naam, Voornaam, Datum en handtekening van de vertegenwoordiger van de therapeut-onderzoeker

____

Een intensief revalidatieprogramma na een centraal neurologische aandoening

III. Aanvullende informatie

Aanvullende informatie over de organisatie van de studie

In onderstaande figuur ziet u een samenvatting van het verloop van de studie. Zie afkortingen van de meetschalen in de tabel op pagina 6-7.

Interventiegroep:
Het eerste deel van de studie bestaat uit de interventie van 3 weken en de pre- en post metingen. Zoals eerder vermeld worden er zowel voor als na de 3 weken intensieve training vragenlijsten afgenomen die gaan kijken naar uw functionaliteit, kwaliteit van leven, persoonlijke doelen en gezondheidseconomische gegevens. Daartussen zit een periode van 3 weken waarin intensief therapie wordt gegeven, een combinatie van conventionele kinesitherapie en hoogtechnologische toestellen. In het tweede deel van de studie wordt u nog tot 9 maanden na de pre-evaluatie opgevolgd. Tijdens die maanden worden uw gezondheidseconomische gegevens en kwaliteit van leven maandelijks bevraagd. Na 9 maanden (T2) worden nogmaals alle vragenlijsten die al bij de voorgaande meetmomenten werden afgenomen (T0&T1), opnieuw afgenomen.

Controlegroep:
De controle groep zet gedurende 9 maanden zijn standaard zorg (vb. 1-2 uur kinesitherapie/week) verder. De meetmomenten zijn gelijkaardig: T0 – T1 – maandelijkse bevraging gezondheidseconomische gegevens/kwaliteit van leven – T2.

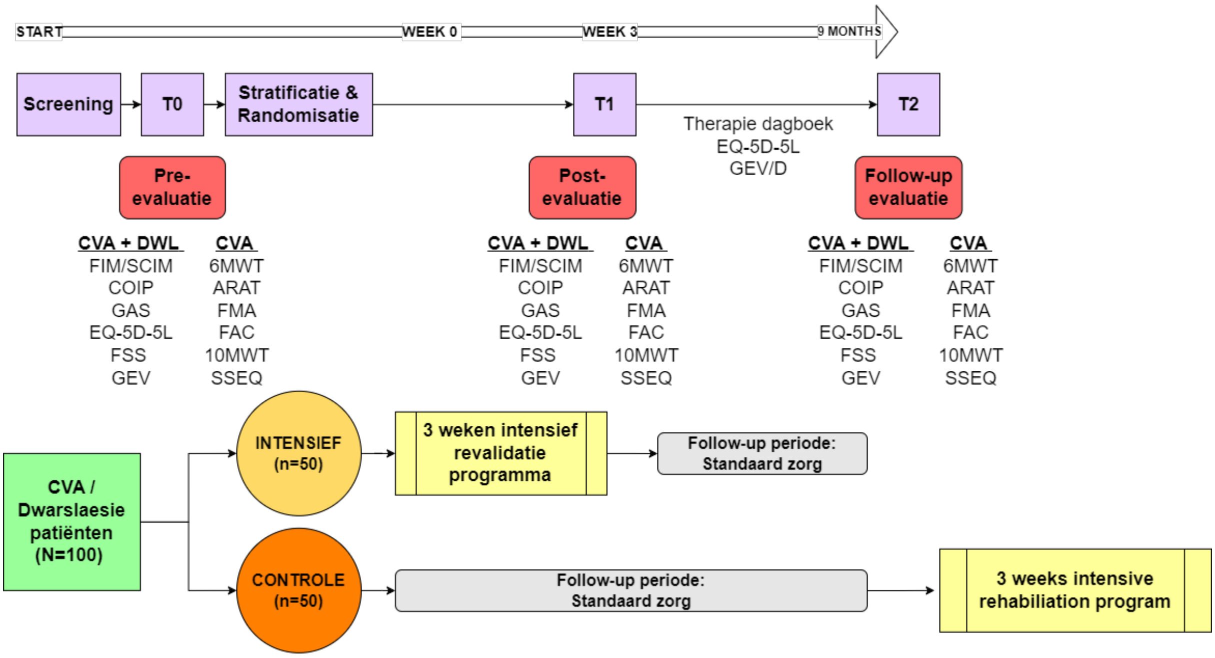

Bescherming en de rechten van de deelnemer aan een klinische studie (bijlage 1)

Ethische comités

Deze studie werd geëvalueerd door meerdere onafhankelijke ethische comités (Ethische Commissie Onderzoek UZ Leuven, Ethische Commissie AZ Herentals en Commissie Medische Ethiek UZ Brussel/ VUB) die een gunstig advies hebben uitgebracht. De ethische comités hebben als taak de personen die aan klinische studies deelnemen te beschermen. Ze controleren of uw rechten als deelnemer aan een studie gerespecteerd worden, of de studie wetenschappelijk relevant en ethisch verantwoord is.

Hierover brengen de ethische comités een advies uit in overeenstemming met de Belgische wet van 7 mei 2004.

U dient het positief advies van de Ethische Comités in geen geval te beschouwen als een aansporing om deel te nemen aan deze studie.

Vrijwillige deelname

Aarzel niet om alle vragen te stellen die u nuttig vindt voordat u tekent. Neem de tijd om er met een vertrouwenspersoon over te praten, als u dit wenst.

U heeft het recht om niet deel te nemen aan deze studie of met deze studie te stoppen zonder dat u hiervoor een reden hoeft te geven, zelfs al hebt u eerder toegestemd om aan deze studie deel te nemen. Uw beslissing zal in geen geval uw relatie met de onderzoeker veranderen, noch de kwaliteit van uw verdere verzorging.

Als u aanvaardt om aan deze studie deel te nemen, ondertekent u het toestemmingsformulier. De onderzoeker zal dit formulier ook ondertekenen en zal zo bevestigen dat hij u de noodzakelijke informatie voor deze studie heeft gegeven. U zult het voor u bestemde exemplaar ontvangen.

Voor uw veiligheid is het wel aanbevolen om de therapeut-onderzoeker op de hoogte te stellen indien u besluit uw deelname aan de studie stop te zetten.

Kosten in verband met uw deelname

Indien u besluit aan deze studie deel te nemen, worden alle onderzoeken en procedures in het kader van de studie door de opdrachtgever betaald, zowel voor de controle- als voor de interventiegroep. Deze onderzoeken en procedures brengen dus geen extra kosten mee voor uzelf, uw naasten of voor uw verzekeringsmaatschappij.

Tijdens de 3 weken intensieve training in AZ Herentals krijgen alle deelnemers tijdens de week overnachtingen gratis aangeboden in de buurt. Er wordt geen gratis verblijf voorzien voor een mogelijke begeleider. Voor de screenings-/meet-/ en therapiemomenten (3 weken intensief programma) wordt gratis parking voorzien. De opdrachtgever zal uw verplaatsingskosten binnen redelijke grenzen vergoeden a.d.h.v. een kilometervergoeding (0.30 euro/km). De nodige financiële gegevens zullen hiervoor worden gevraagd.

Het studieteam zal meer uitleg geven over de praktische uitvoering.

Vertrouwelijkheidgarantie

Uw deelname aan de studie betekent dat de therapeut-onderzoeker gegevens over u verzamelt en dat de opdrachtgever van de studie die gebruikt voor onderzoek en in het kader van wetenschappelijke en medische publicaties.

De verwerking van uw persoonsgegevens is noodzakelijk om de wetenschappelijke onderzoeksdoeleinden zoals hierin te kunnen realiseren. Het uitvoeren van academische onderzoek behoort tot wettelijke opdrachten van KU Leuven als opdrachtgever. KU Leuven dient immers wetenschap en onderwijs in het algemeen belang te ondersteunen. KU Leuven verduidelijkt u graag

dat de noodzakelijkheid van de verwerking voor het uitvoeren van wetenschappelijk onderzoek en dit als taak van algemeen belang, de wettelijke toelatingsgrond vormt op basis waarvan KU Leuven in het kader van dit onderzoek uw gegevens verwerkt. Daarnaast is KU Leuven onderhevig aan specifieke wettelijke verplichtingen die de verwerking van uw gegevens mogelijks noodzakelijk maken in het kader van veiligheidsrapportering (zoals bijvoorbeeld het melden van bijwerkingen aan toezichthoudende overheidsinstanties).

Uw gegevens zullen worden verwerkt overeenkomstig de Europese Algemene Verordening inzake Gegevensbescherming (AVG) en de Belgische Wetgeving betreffende de bescherming van natuurlijke personen met betrekking tot de verwerking van persoonsgegevens. Als opdrachtgever van het onderzoek, is KU Leuven de verwerkingsverantwoordelijke van uw persoonlijke gegevens die verwerkt worden in het kader van het onderzoek.

U hebt het recht om aan de therapeut-onderzoeker te vragen welke gegevens hij/zij over u heeft verzameld en waarvoor ze gebruikt worden in het kader van de studie. Deze gegevens hebben betrekking op uw huidige klinische situatie maar ook op uw medische voorgeschiedenis en op de resultaten van onderzoeken die werden uitgevoerd voor de behandeling van uw gezondheid volgens de geldende zorgstandaard. U hebt het recht om deze gegevens in te kijken en om verbeteringen te laten aanbrengen indien ze foutief zouden zijn[1]. Uw gegevens zullen 25 jaar bewaard worden.
De therapeut-onderzoeker is verplicht om deze verzamelde gegevens vertrouwelijk te behandelen. Dit betekent dat hij zich ertoe verbindt om uw naam nooit bekend te maken in het kader van een publicatie of een conferentie en dat hij uw gegevens zal coderen (uw identiteit zal worden vervangen door een identificatiecode in de studie) voordat hij/zij ze doorgeeft aan de beheerder van de databank (KU Leuven, Departement Revalidatiewetenschappen, Tervuursevest 101, bus 1501, 3001 Leuven).
De therapeut-onderzoeker en zijn team zullen gedurende de volledige klinische studie de enige personen zijn die een verband kunnen leggen tussen de overgedragen gegevens en uw medisch dossier[2].
De overgedragen persoonlijke gegevens omvatten geen combinatie van elementen waarmee het mogelijk is u te identificeren[3].

De door de opdrachtgever aangestelde beheerder van de onderzoeksgegevens kan u niet identificeren op basis van de overgedragen gegevens. Deze persoon is verantwoordelijk voor het verzamelen van de gegevens die door alle therapeuten-onderzoekers die deelnemen aan de studie zijn verzameld en voor de verwerking en de bescherming van die gegevens in overeenstemming met de Belgische wet betreffende de bescherming van de persoonlijke levenssfeer.

Om de kwaliteit van de studie te controleren, kan uw medisch dossier worden ingekeken door personen die gebonden zijn aan het beroepsgeheim zoals vertegenwoordigers van de ethische comités, van de opdrachtgever van de studie of een extern auditbureau. Dit kan enkel gebeuren onder strikte voorwaarden, onder de verantwoordelijkheid van de therapeut-onderzoeker en onder zijn/haar toezicht (of van één van zijn/haar onderzoeksmedewerkers).

De (gecodeerde) onderzoeksgegevens kunnen doorgegeven worden aan Belgische of andere regelgevende instanties, aan de ethische comités, aan andere therapeuten/artsen en/of instellingen die samenwerken met de opdrachtgever. Deze artsen en/of instellingen kunnen zich situeren in België en in andere landen waar de normen inzake de bescherming van persoonsgegevens verschillend of minder strikt kunnen zijn . Dit gebeurt dan steeds in gecodeerde vorm zoals hierboven uitgelegd.

Uw toestemming om aan deze studie deel te nemen betekent dus ook dat uw gecodeerde medische gegevens gebruikt worden voor doeleinden die in dit informatieformulier staan beschreven en dat ze worden overgedragen aan bovenvermelde personen en/of instellingen.

[1] Deze rechten zijn bepaald door de Europese Algemene Verordening Gegevensbescherming (AVG), de Belgische Wetgeving betreffende de bescherming van natuurlijke personen met betrekking tot de verwerking van persoonsgegevens en door de wet

van 22 augustus 2002 betreffende de rechten van de patiënt.
[2] De wet verplicht om voor klinische studies uw dossier gedurende 20 jaar te bewaren.
[3] De database met de resultaten van de studie zal dus geen elementen bevatten zoals uw initialen, uw geslacht en uw volledige geboortedatum (dd/mm/jjjj).

Indien u uw toestemming tot deelname aan de studie intrekt, zullen de gecodeerde gegevens die al verzameld waren vóór uw terugtrekking, bewaard worden. Hierdoor wordt de geldigheid van de studie gegarandeerd. Er zal geen enkel nieuw gegeven aan de opdrachtgever worden doorgegeven.

Indien u vragen hebt over hoe wij uw gegevens gebruiken of uw recht op inzage, correctie, eventueel stopzetting van de verdere verwerking wil uitoefenen, dan kan u hiervoor steeds terecht bij uw therapeut-onderzoeker op volgend emailadres: […]. Indien u naderhand nog bijzondere aandachtspunten heeft of klacht wenst neer te leggen, kan u terecht bij het privacy team van de KU Leuven op […].

Tot slot, indien u een klacht heeft over de verwerking van uw gegevens, kunt u contact opnemen met de Belgische toezichthoudende instantie die toeziet op de naleving van de grondbeginselen van de bescherming van persoonsgegevens.

De Belgische toezichthoudende instantie heet:
Gegevensbeschermingsautoriteit (GBA)
Drukpersstraat 35,
1000 Brussel
Tel. […]
e-mail: […]
Website: […]

Verzekering

Elke deelname aan een studie houdt een risico in, hoe klein ook. De opdrachtgever is - ook indien er geen sprake is van fout - aansprakelijk voor de schade die de deelnemer of in geval van overlijden zijn/haar rechthebbenden, oplopen en die rechtstreeks of onrechtstreeks verband houdt met diens deelname aan de studie. U moet hiervoor dus geen fout aantonen. Hiervoor heeft de opdrachtgever een verzekeringscontract afgesloten ([…])[4].

Indien de therapeut-onderzoeker van mening is dat er een verband met de studie mogelijk is (er is geen verband met de studie bij schade ten gevolge van het natuurlijke verloop van uw ziekte of ten gevolge van gekende bijwerkingen van uw standaardbehandeling), zal hij/zij de opdrachtgever van de studie op de hoogte stellen die de aangifteprocedure bij de verzekering zal starten. Deze zal, indien zij het nodig acht, een expert aanstellen om een oordeel uit te spreken over het verband tussen uw nieuwe gezondheidsklachten en de studie.

In het geval van onenigheid met de therapeut-onderzoeker of met de door de verzekeringsmaatschappij aangestelde expert, en steeds wanneer u dit nodig acht, kunnen u of in geval van overlijden uw rechthebbenden de verzekeraar rechtstreeks in België dagvaarden .

De wet voorziet dat de dagvaarding van de verzekeraar kan gebeuren ofwel voor de rechter van de plaats waar de schadeverwekkende feiten zich hebben voorgedaan, ofwel voor de rechter van uw woonplaats, ofwel voor de rechter van de zetel van de verzekeraar.

[4] In overeenstemming met artikel 29 van de Belgische Wet inzake experimenten op de menselijke persoon (7 mei 2004).

STROKE REHABILITATION RESEARCH TEAM
Tervuursevest 101 – bus 1501
3001 LEUVEN, België
