# Supplementary material for: Impact of a Personalized, High-Dose, Intensive Motor Rehabilitation Program, Integrating Advanced Technology for Adults With Central Neurological Conditions (INTeRAcT): Protocol for a Single-Blind Randomized Controlled Trial With a Clinical, Health Economic, and Process Evaluation
Source: JMIR Res Protoc. 2026 May 4;15:e93234. doi: 10.2196/93234 (PMC13151458; doi:10.2196/93234)
Supplement: Multimedia Appendix 2 — Overview of demographic, socioeconomic, and pathology-specific variables. [file resprot-v15-e93234-s002.pdf]

DEMOGRAPHIC AND SOCIOECONOMIC VARIABLES
**Self-identified sex** (M, F, X)
**Age** (years)
**Living arrangement**<br>(living with partner/and or kids, living alone, living with family, residential care facility or other)
**Age of cohabiting children** (years)
**Marital status**<br>(married, legally cohabiting, divorced, unmarried, single, widowed, or other)
**Education level**<br>(No formal education, primary school, lower or upper secondary education, post-secondary non-tertiary education, college graduate, university bachelor's degree, university master's degree, PhD/Doctorate, or other)
**Characteristics of (previous) job**<br>(job title/description; self-employed or employee)
**Current employment status**<br>(full-time, part-time, unemployed, retired, stay-at-home caretaker/homemaker, on disability)
**Self-reported monthly income** (€)

Demographic and socioeconomic data collection. *Overview of the demographic and socio-economic variables and associated outcomes (indicated in parentheses) collected at baseline assessment (T0). M = male, F = Female, X = undifferentiated.*

PATHOLOGY-SPECIFIC VARIABLES	
STROKE	SPINAL CORD INJURY
**Time since onset** (days)	
**Type of stroke** (ischemic, haemorrhagic, mixed)	**ASIA Impairment Scale classification** (A, B, C, D)
**Lesion side**<br>(left or right brain hemisphere, both or other)	**Classification of injury**<br>(complete or incomplete; paraplegia or tetraplegia / quadriplegia)
**Lesion location** (descriptive)	
**Motor hemiparesis** – **upper/lower limb** (left, right, both, not affected)	
**Dominant hand/leg** (left, right, unknown)	
**Relevant medical history** (descriptive)	
**Usual care** (content and frequency of physiotherapy and/or occupational therapy)	
**Self-reported comorbidities** (cardiovascular, respiratory, ear/nose/throat, gastrointestinal, urogenital, musculoskeletal, neurological, psychiatric, and endocrine/metabolic disorders)	
**Perceived impact of comorbidities on daily life**<br>(assessed on a 5-point scale ranging from “no comorbidity” to “very severe, life-threatening”)	

Pathology-specific data collection. *Overview of pathology-specific variables and associated outcomes (indicated in parentheses) collected at baseline assessment (T0).*
