# Supplementary material for: Impact of a Personalized, High-Dose, Intensive Motor Rehabilitation Program, Integrating Advanced Technology for Adults With Central Neurological Conditions (INTeRAcT): Protocol for a Single-Blind Randomized Controlled Trial With a Clinical, Health Economic, and Process Evaluation
Source: JMIR Res Protoc. 2026 May 4;15:e93234. doi: 10.2196/93234 (PMC13151458; doi:10.2196/93234)
Supplement: Peer Review Report 1 — Peer review report by the College of Medical Directors, the National Institute for Health and Disability Insurance (NIHDI/RIZIV, Belgium). [file resprot-v15-e93234-s005.pdf]

Subscribe to DeepL Pro to translate larger documents.
Visit www.DeepL.com/pro for more information.

Subject Research protocol To Walk Again/KULeuven ihkvv art.56
| Effect of an intensive rehabilitation programme integrating technology for adults with central neurological disease |

Responses to the lecture by the College of Medical-Directors, online meeting dated 1 April 2021, **with proposal for adaptation**

Prof. Geert Verheyden
Griet De Ceuster

To Walk Again - Post rehabilitation, supported by Prof. Geert Verheyden (KU Leuven, penholder for the research project) thanks the College for the reading of and comments on the research project, and for the opportunity to discuss it, in consultation with the KCE.

First, the general research framework is given below, followed by responses to the College's comments. Finally, the modified research project is attached in Appendix 1.

This research project falls within the objective of evaluating the **use of height technology in rehabilitation**. By height technology we mean advanced technological rehabilitation equipment for lower limbs (e.g. step robot) and upper limbs (e.g. arm robot). Rehabilitation should be understood as the therapy given at any time post pathology and is therefore not only linked to a 'subacute' stay in a rehabilitation centre. Rehabilitation is therefore also meant as the therapy that patients receive after a rehabilitation stay, in other words in the 'chronic' phase after a condition when patients have returned to the home situation.

<u>Research framework</u>
The research framework starts from the following three elements:

- Rehabilitation after central neurological disease is meaningful and necessary **at any time after the condition**. Immediately after the condition there is a window for spontaneous recovery, which is presumably for e.g. stroke to 6-8 weeks post insult (also checked by Dr Roothans during the online discussion). In this window there is the possibility of biologically-induced plasticity or, in other words, recovery of brain function. What impact rehabilitation has on this (subacute) recovery is not sharply described in the literature. It is known that in the first weeks to months patients demonstrate the most recovery[1]. However, this recovery is highly variable and this heterogeneity makes research in this early (subacute) phase a complex issue. Rehabilitation with a certain intensity is also anticipated in this early phase. A

[1]Verheyden G, Nieuwboer A, De Wit L, et al. Time course of trunk, arm, leg, and functional recovery after ischemic stroke. Neurorehabil Neural Repair. 2008 Mar-Apr;22(2):173-9.

significantly higher intensity in a very early phase post pathology was also already found to have less good effects than a lower-intensity programme.[(2) ,3]

- Rehabilitation brings the patient to a certain level of functioning, but this is not the pre-pathology level for hospitalised patients who are subsequently admitted to a rehabilitation programme. Thus, limitations remain when patients are discharged from rehabilitation to the home setting. These patients are then entitled to outpatient therapy in the chronic phase, but at a much lower frequency than during rehabilitation. Moreover, it is known in the literature that **patients in a chronic phase, in the long term, lose functionality** and thus evolve to a lower level of activity[4].
  Here, related problems are also observed, often linked to reduced mobility and activity such as bedsores, osteoporosis, use of antidepressants, cystitis, and others. So more intensive **rehabilitation in a chronic phase is appropriate**. Spontaneous recovery is not present here; in fact, improvement here is characterised by exercise-induced plasticity, which amounts to an improvement in functionality through increases in strength, endurance, flexibility and coordination, among others. In general, the more intensive work can be done, the more improvement can be seen.
- Although studies with a positive effect are seen in the literature about the effect of rehabilitation, these are more likely to be studies with a small patient number. Looking at large, phase III studies, the results are disappointing and sometimes even negative. This is partly due to the fact that the study design emphasises one aspect of rehabilitation, such as strength training for spinal cord injury patients[5]or robot training for stroke[6]. Research already showed that the **combination of therapy and height technology** is **useful** in a subacute phase after pathology[7], and an important question is therefore **whether this applies to the chronic phase**. There is recent research by Ward and colleagues from London offering a 90-hour therapy programme for stroke patients showing significant and clinically relevant progress post intervention and in the long-term.[8]However, this was a cohort study with no control group.

In summary, therefore, further research needs to be done on rehabilitation in the subacute phase; how patients can improve more, but does the variable spontaneous recovery in this phase make this research complex. In the chronic phase, with permanent impairments in a large group of patients with central neurological diseases, we see patients losing functionality and the question is how therapy and height technology can lead to additional clinical gains in long-term functionality, for patient and society.

---

[2]Dromerick AW, Lang CE, Birkenmeier RL, et al. Very Early Constraint-Induced Movement during Stroke Rehabilitation (VECTORS): A single-center RCT. Neurology. 2009 Jul 21;73(3):195-201.
[3]AVERT Trial Collaboration group. Efficacy and safety of very early mobilisation within 24 h of stroke onset (AVERT): a randomised controlled trial. Lancet. 2015 Jul 4;386(9988):46-55.
[4]Meyer S, Verheyden G, Brinkmann N, et al. Functional and motor outcome 5 years after stroke is equivalent to outcome at 2 months: follow-up of the collaborative evaluation of rehabilitation in stroke across Europe. Stroke. 2015 Jun;46(6):1613-9.
[5]Chen LW, Glinsky JV, Islam MS, et al. The effects of 10,000 voluntary contractions over 8 weeks on the strength of very weak muscles in people with spinal cord injury: a randomised controlled trial. Spinal Cord. 2020 Aug;58(8):857-864.
[6]Rodgers H, Bosomworth H, Krebs HI, et al. Robot assisted training for the upper limb after stroke (RATULS): a multicentre randomised controlled trial. Lancet. 2019 Jul 6;394(10192):51-62.
[7]Pohl M, Werner C, Holzgraefe M, et al. Repetitive locomotor training and physiotherapy improve walking and basic activities of daily living after stroke: a single-blind, randomised multicentre trial (DEutsche GAngtrainerStudie, DEGAS). Clin Rehabil. 2007 Jan;21(1):17-27.
[8]Ward NS, Brander F, Kelly K. Intensive upper limb neurorehabilitation in chronic stroke: outcomes from the Queen Square programme. J Neurol Neurosurg Psychiatry. 2019 May;90(5):498-506.

Following comments and discussion, we **propose** a **modified research project** with the following core elements:

- A **randomised controlled trial in the chronic phase** after central neurological disease (stroke or paraplegia), i.e. when the patient is back in the home situation. From the discussion on 1 April, we infer that a focus to this phase can be understood and will provide new outcomes for intensive rehabilitation in the chronic phase after stroke and spinal cord injury.
- **Primary objective is to allow patients to make significant and clinical progress after intensive rehabilitation** through the combination of therapy and height technology. Secondary, long-term preservation will be evaluated as well as a health economic analysis.
- The **combination of therapy and height technology** is maintained. High-technology delivers exercise at a high intensity, allowing more progress. This still requires trained staff as verified by Dr De Koekelaere. This expertise is present in To Walk Again. In addition to high-technology, (regular) therapy is required to make the transfer from progress in movement to **improvement in functionality**.
- Both pathological populations (**stroke and paraplegia**) are retained in the protocol because functionality in both target populations is limited by similar symptoms due to central neurological disease, and the approach for both target populations should be a combination of therapy and height technology. This combination provides a transfer of improvement through height technology to functionality. Both patient groups are also dominant in the setting where the study continues, To Walk Again in Herentals.
- A rehabilitation protocol where **90 hours of therapy** are given spread over 3 weeks.
- Focusing on the later phase may increase the number of patients for this study, and we aim to recruit a total of **100 patients** for this study. The benefit of a multicentre study was indicated by Dr Hulstaert (KCE). Our study will initially do recruit patients from To Walk Again and Hospital Geel, who will continue to receive their regular (chronic) therapy at both sites. The excess therapy up to 90 hours will be given at To Walk Again for both groups. In addition, we want to explore recruiting chronic patients from the wider Hospital Kempen network. To Walk Again has a large patient base, 85% of whom are stroke or paraplegic patients. There is also a waiting list. With To Walk Again, Geel, possibly the other regional hospitals and local networks (GP/kinesitherapy practices), the number of 100 patients to be recruited in two years seems achievable.
- The control group will still be a '**waiting list control group**', namely patients who will receive the 3-week intensive rehabilitation programme after 9 months (after inclusion in the study). In our opinion, this remains an important aspect to maintain the motivation of included patients.
- The chronic phase study will also provide the greatest **contrast** between the experimental (90 hours of therapy in three weeks) and control group (3-6 hours of therapy in three weeks on average). This contrast also gives rise to our hypothesis that for this comparison, a clear, greater improvement in functionality can be expected in the experimental group.

In summary, this is presented in **PICO format** as follows:

**P(atient)**	**Is in patients after stroke or paraplegia staying at home after rehabilitation admission**
**I(ntervention).**	A 3-week (90-hour) intensive rehabilitation programme of therapy and height technology
**C(ontrol)**	Compared to the regular therapy patients normally receive in this chronic phase
**O(utcome)**	Safe and meaningful to achieve clinical progress in functionality and quality of life, also in the long term with health economic benefits?

Responses to the Board's comments

The following is a summarised response to the College's comments and questions and comments raised additionally during the online meeting on 1 April 2021.

"The College questions the objectives of the study. It is usually assumed that rehabilitation is best started as soon as possible after the "event" from which the need for rehabilitation arose and possibly already started while the patient is still in intensive care. What the study seeks to demonstrate (*namely that intensive brief therapy can be as successful as time-spread less intensive therapy; that late intensive therapy can still be useful and produce results*) is, in the College's view, already described in the literature. The College therefore questions whether this study will provide new knowledge. If the study does not really provide new knowledge, the only effect will be to obtain insurance intervention through the study for the use of high-tech devices and for more intensive rehabilitation."

Answer: About the time after the event, also during the online discussion made by Dr Van Elshocht, we refer to the research framework. We would also like to refer back to the research framework for the innovative aspect. Given the comments and online discussion, we thus focus on:

- *The chronic phase after central neurological disease, as this is where we still see residual impairment in both populations, caused by similar symptom moment (including muscle weakness, spasticity, loss of endurance, lack of coordination). The progress we seek comes from exercise-induced plasticity.*
- *An intensive package of rehabilitation in combination therapy and altitude technolology what not yet proven in a randomised controlled trial with enough patients. The sharpened focus in our adapted protocol (n=100) would make that the case.*

We certainly agree that early intensive rehabilitation is also useful, but at a higher intensity than currently offered in rehabilitation centres. Therefore, we would like to propose the research project initially proposed as ongoing within UZ Leuven (therapy and height technology in subacute phase) as a project idea for the next KCE trials submission, as indicated by Dr Hulstaert (KCE) during the discussion. This is because, as a team, we also still support rehabilitation research with

therapy and height technology for this phase. With this study, we also believe we can move towards a multicentre study, which is recognised to be of benefit. In this study application, To Walk Again will also be involved because we want to start from this multicentre 'rehabilitation hubs' that can provide therapy+high technology for the sub-acute phase. To Walk Again with Hospital Network Kempen is seen as such a 'hub'.
For the current study, we initially want to start from To Walk Again as a therapy setting because, as a centre, this setting is quite unique in Flanders, Belgium and beyond. There is currently no other organisation or institution in Belgium that offers therapy in this way and we therefore propose to initially start the study in this centre. As already indicated, it is true that we will also include patients receiving chronic therapy at Ziekenhuis Geel, so in that respect two centres are already involved in the study. And we want to extend recruitment to the whole Hospital Kempen network.

"The Board is of the opinion that a quality study based on the proposed research project is not possible because:

- The number of patients in the study is too small

Answer: In our initial protocol, we assumed a power calculation and the number of patients would have been sufficient for a clinically relevant effect of 16%. It is true that hypothetical assumptions were used for this calculation, based on literature, but not on pilot data, as also discussed with Dr Hulstaert (KCE). It is also acknowledged that the number of participants per study was small compared to typical phase III studies. By focusing on one setting in the modified protocol, we can increase the number of patients to 100 and indicate with greater certainty that we will find a difference for patients and society.

- "The included patients are too heterogeneous:
    - o the patients suffer from diverse pathologies that may not be taken together in one study,
    - o the period elapsed since the "event" occurred from which the need for rehabilitation arose is far too broad.

  That all kinds of data about the patients, their condition and their rehabilitation will be recorded in detail, so that when the results are analysed it can be checked whether the groups are comparable or not, does not provide enough guarantees for a quality study. If, on the basis of this analysis, the study population were actually found to be too heterogeneous to draw conclusions from the study - as the College fears - the budget for this research project has not been put to good use."

Answer: We still believe that we can include both patient populations in one study, as indicated above because we are not going to treat pathology but impairments leading to loss of functionality, as discussed on response from Dr Roothans. These are very similar for both these populations (stroke and paraplegia). Even in current practice, the approach to therapy (treating limitations and transferring to increased functionality) is the same for these groups.

Besides, each pathology is also different in itself, and a population of chronic stroke patients is by definition not homogeneous. The answer to this remains taking a detailed picture of the patient with collection of data at study entry so that if there is a difference in these factors, this can be included as an influencing factor. This therefore remains the intention.

As for the period elapsed, the study at a later stage reflects clinical reality. In that respect, our study is pragmatic. What is important, however, is that our analysis will obviously look at the progress patients make for functionality.
Since progress is the difference between final measurement and initial measurement, this controls for variability in initial status. As described above, our analysis also takes into account the influencing factors. Finally, as indicated in our research framework, we want to reiterate that time post pathology is only a factor relevant to spontaneous-induced plasticity in the literature, but that is not the plasticity we are aiming for in this study.
Here we are talking about exercise-induced plasticity, in other words improvement through exercise, and this is independent of time after event in a chronic phase, both for stroke and for paraplegia.

"The Board understands that with the 2 centres involved in the study, it is not possible to delineate the target population more and also include more patients in the study, because the number of patients who would meet the conditions for inclusion in the study would then be too small in the two centres. However, the fact that there are not enough patients in the two centres to arrive at a sufficiently large, homogeneous study population is no reason for the Board to then conduct a study in a study population that is too small and heterogeneous."

Answer: With the proposed focus on a larger single-stage study, we believe we are responding to these comments. This would allow us to focus the commitment of resources on a larger patient population in two centres (To Walk Again and via Geel), and possibly two additional centres in the wider Kempen region.

"One of the objectives of the study is to demonstrate that late intensive therapy can still be useful. In this regard, however, the Board notes that the last evaluation moment falls immediately after late intensive therapy. Consequently, the long-term effects of late intensive therapy are not examined with this study."

Answer: We believe this refers to the study that would have been started in UZ Leuven, but with an adapted protocol with a study only in To Walk Again, this comment no longer seems applicable. However, it is true that in To Walk Again we will still offer the control group (after 9 months) the intensive rehabilitation package because this is a waiting list control group. It is true that the study stops after this rehabilitation for the control group with the last measurement. The long-term effect is obviously integrated into our study design because we follow up patients in both groups up to 9 months after inclusion.

"The research project shows that these are in fact 2 different studies because the 2 centres rehabilitate different patient populations and the rehabilitation offer of the 2 centres is different

is. The period elapsed since the "event" occurred from which the need for rehabilitation arose is also different for the 2 centres. It is therefore not possible to compare the results of the 2 centres. The research project shows that such a comparison of the results of the 2 centres is also not intended. Consequently, in practice, the studies are 2 similar studies. But in that case, the objectives of each of those studies should be explained and, for each of them, it should be justified what new knowledge it will provide."

Answer: This is certainly correct as indicated by the Board. With the modified project directed towards the centre To Walk Again, there has been more focus in the project and the objective has been sharpened, as indicated in the PICO model on page 4.

Also, College President Dr De Winne asked whether the research project had already received a positive opinion from an ethics committee. This is not yet the case. The protocol has also not yet received final approval, and comments and discussions have led to important modifications. A submission will therefore wait for a positive decision. However, the documents (including ICF) have already been prepared so that a submission can follow a positive opinion.

--

Appendix 1. Adapted research protocol

1. Application for ethical approval

The application and all additional documents for the study will be brought to the Ethics Committee for Research at UZ/KULeuven as this will act as the ethics committee.

2. Recruitment of participants for the RCT

Participants with central neurological disorders especially stroke and paraplegia will be recruited. We expect to enroll 100 patients in total (4 patients per month over a 25-month period).

To Walk Again has a patient base of 99 people, 85% of whom are patients with stroke or paraplegia. There is also a waiting list. With To Walk Again, Hospital Geel, possibly the other regional hospitals (Mol and Turnhout) and local networks (GP
/kinesitherapy practices), the number of 100 patients seems achievable.

3. Inclusion and exclusion criteria

Requirements for participation include: (i) a diagnosis of central neurological disease made by a specialist, no longer receiving daily rehabilitation under a convention, living at home and with a maximum recovery of 85% of independence in daily functioning (see primary outcome measure 'functionality' how this will be evaluated). Conditions may include stroke or paraplegia; (ii) unilateral or bilateral upper and/or lower limb weakness; (iii) clinical ability to follow exercise therapy with available technology;
(iv) an age of >18 years; and (v) sufficient cognitive ability to understand the games and provide informed consent.

Participants will be excluded from the study if they: (i) have other neurological or musculoskeletal conditions that may affect the protocol; (ii) no ability to initiate movements against gravity in the upper and lower limbs; (iv) severe visual impairment and (v) severe communication, cognitive and language formation problems that would interfere with the intervention and measurement process.

Inclusion criteria for the specific conditions

Stroke

Participants must have received a diagnosis of stroke for the first time, have been diagnosed at least 3 months ago, and live at home.

Transverse stroke

Participants have a classification A, B, C or D on the American Spinal Injury Association (ASIA) measurement scale, which refers to patients with complete or incomplete impairment.

4. Design and Data collection

a) ***Design***

First, demographic data and baseline characteristics of patients such as age, gender and preferred hand, as well as time post pathology and cognitive

condition. Specifically for people with stroke, lateralisation of symptoms, type and location of stroke will be retrieved. People with spinal cord injury will be classified using the ASIA scale.

Participants will be stratified for the condition they have; stroke or spinal cord injury. Thus, we obtain an equal distribution of condition between the experimental and control groups of the RCT.

The included patients (n=100) will then be randomly assigned using a computer system to one of 2 groups, namely the experimental group 'INTENSIVE' (n=50) or the control group 'CONTROL' (n=50). Each group will be measured immediately after baseline randomisation (T1), then after a 3-week period (T2) and a follow-up measurement (T3) after 9 months (Figure 1). The intensive group will receive a 90-hour intensive rehabilitation programme within three weeks after baseline. The wait-list control group will receive this 3-week intensive rehabilitation after the follow-up measurement 9 months after baseline measurement. Finally, a final measurement (T4) will follow after this three-week period. Behavioural outcome measures will be collected at these four time points, as well as measurements to calculate cost-effectiveness and the latter in collaboration with a centre of expertise in this field. A visual overview of the study design is depicted in Figure 1.

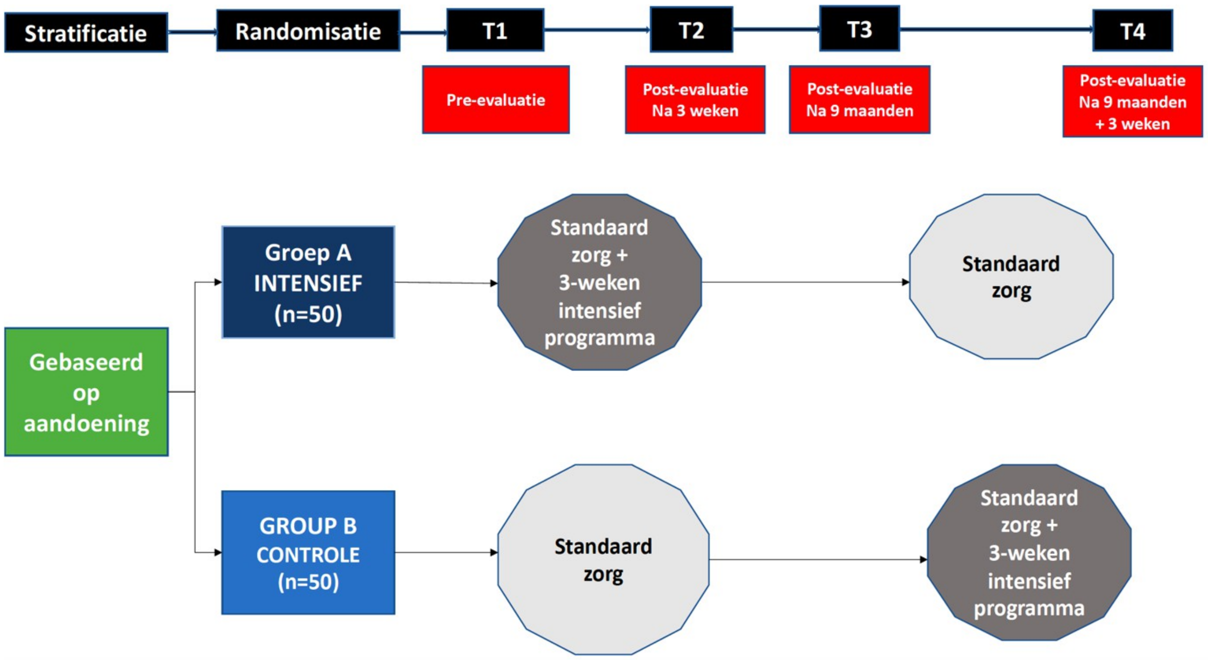

Figure 1: Design of the RCT

b) ***Evaluation of clinical outcome measures: T1, T2, T3 and T4***

All outcome measures will be measured by an independent and trained assessor blinded to group assignment. The primary outcome measure 'functionality' will be the Functional Independence Measure (FIM), which measures independence in daily functioning after stroke. The FIM consists of 18 items designed to ascertain the extent to which a person needs assistance in performing activities of daily living safely and efficiently. The activities consist of a number of basic skills related to self-care,

pelvic floor function, transfers, gait, communication and social abilities. The FIM has acceptable reliability across a wide range of gauges, settings and patients. The FIM has been found valid in people with various neurological conditions including stroke. In people with spinal cord injury, the Spinal Cord Independence Measure (SCIM) will be used, which encompasses the following areas of independence in daily functioning: self-care, breathing, pelvic floor control and mobility.

To compare our patients within one centre, we work with % of improvement for these metrics collected. For both stroke and spinal cord injury patients, the primary outcome measure is a measurement of functionality, so it has the same construct for both populations. Therefore, the proposal to express these scores in % and therefore be able to take both pathologies together.

The secondary outcome measures will relate to goal setting, limitations, activities and participation, based on the International Classification of Functioning, Health and Disability (ICF). The Goal Attainment Scaling (GAS) is a measurement scale that quantifies the achievement of formulated goals and is used in rehabilitation settings. The GAS can and will be used for both stroke and spinal cord injury.

The Canadian Occupational Performance Measure, which measures assessment of one's own functioning ability in daily life over time, and the EQ-5D which measures quality of life will also be included. These measurements have also been found to be valid and reliable in people with central neurological disorders. Finally, the Neurological Fatigue Index will be used so that participants can indicate the extent to which they experience fatigue. These will also be used for both stroke and paraplegia.

5. **Intervention**

Participants in the control group will receive the normal therapy offered as standard according to the nomenclature in the chronic phase. The normal care obtained will be carefully documented for all included patients.

The patients in the intensive group will also continue to receive normal standard therapy, but will additionally receive a 3-week intensive programme on 5 days a week for up to 6 hours a day as shown in Figure 2.

The participants in the intensive group receiving therapy in Herentals (these are also the patients from Geel and possibly other hospitals that will be included in this study) will be offered overnight accommodation during the 3 weeks, near To Walk Again.

As indicated, we will measure the rehabilitation the patients receive within standard care. We will do this from the start of the study (T1) to T4. We will do this by:

- Using records of treatments that happen within the centre.

- Using a therapy diary that we ask patients to fill in, on a weekly basis. In it we ask to indicate (by patient or partner/caregiver) which therapy was followed and the duration of treatment.

This will give us an overall view of rehabilitation from T1 to T4. We can then show that this care is not different between the groups, or if there is a difference, include this difference in the analysis of the data in order to interpret results meaningfully anyway.

<table>
<tr><th></th><th>Monday</th><th>Tuesday</th><th>Wednesday</th><th>Thursday</th><th>Friday</th></tr>
<tr><td>09.30</td><td colspan="5">Cardiovascular fitness training</td></tr>
<tr><td>10.00</td><td rowspan="2">Normal care</td><td rowspan="2" colspan="4">Upper limb therapy</td></tr>
<tr><td>10.30</td></tr>
<tr><td>11.00</td><td rowspan="2" colspan="5">Therapy upper limb+ technology</td></tr>
<tr><td>11.30</td></tr>
<tr><td>12.00</td><td rowspan="2" colspan="5">Lunch</td></tr>
<tr><td>12.30</td></tr>
<tr><td>13.00</td><td rowspan="2" colspan="2">Therapy lower limb</td><td rowspan="2">Normal care</td><td rowspan="2" colspan="2">Therapy lower limb</td></tr>
<tr><td>13.30</td></tr>
<tr><td>14.00</td><td rowspan="2" colspan="5">Therapy lower limb+ technology</td></tr>
<tr><td>14.30</td></tr>
<tr><td>15.00</td><td rowspan="2" colspan="5">Individual training according to objectives</td></tr>
<tr><td>15.30</td></tr>
<tr><td>16.00</td><td colspan="5">Self-management</td></tr>
</table>

Figure 2: Example of an intensive programme for chronic patients. For these patients, the therapy given is currently limited to, in this example, two 1-hour sessions per week (indicated in green).

The intensive rehabilitation concept is goal-oriented; together with the team and the patient, specific goals are set, which are evaluated after three weeks. The content of the programme is formed by strengthening physical fitness through fitness training, therapy for upper limb using high-technology equipment, therapy for lower limb using high-technology equipment, individual training strongly linked to personal goals and making the transfer to functionality, and the intiation of self-management to promote self-efficacy in the patient after the end of the programme.

Participants in the intensive group will start and end the day with cardiorespiratory fitness training. Depending on the setting, this includes treadmill training, normal gait training, bicycle ergometer, stepping, sports activities and outdoor activities such as swimming, ball games, cycling and walking. In Herentals, a wide variety of equipment is available such as a 'chest press', 'peck deck', 'ski fit', 'shrug', 'push-up' and 'pull-down'. Supervising therapists take care of the settings of these devices, which are reviewed and adjusted for each patient. In general

overall, the aim is to enhance self-efficacy and motivation by gradually reducing assistance. Cardiorespiratory fitness training will serve as a warm-up at the beginning of the day.

The second block of the day will focus on upper limb therapy. Physiotherapy sessions of the arm and hand will include mobilisations, analytical and coordination exercises, task training with repetition, fen unctional exercises. These will be complemented by available technologies used in rehabilitation care. In Herentals, for example, the Gloreha© robot will be used for passively or actively assisted finger mobilisation and proprioception training.

In the afternoon, the focus will be on lower limb rehabilitation. Similar to the upper limb time block, passive/active/assisted/independent therapy will be g i v e n with and without the help of technology. Depending on the disability, therapy will include stepping with or without a robotic device, mobilisations, strength training, analytical exercises, functional exercises, fall prevention training, and coordination training. Both upper and lower limb therapy will be tailored to the patient and adapted to patient-specific goals and degree of restriction. Progression is built in throughout the weeks.

Therapist and patient together create an individual training according to the specific goals at the end of the day after which a self-management programme is concluded, laying the foundation for developing self-efficacy in the patient.

Participants in the control group will receive an identical 3-week programme after the follow-up measurement at 9 months. They are thus a wait-list control group and this to maintain motivation to stay in the study.

6. **Statistical analysis and cost-effectiveness**

An evaluation of the feasibility of the intensive programme will be conducted as well as a statistical comparison between the intensive and control group with a mixed models analysis to evaluate the differences in behavioural measures between baseline, post intervention and after follow-up. A cost-effectiveness analysis will also be conducted based on the comparison between intensive versus control group within the 9-month time horizon, based on a cost-utility analysis (effects measured as utility via EQ-5D). The analysis includes direct medical costs via billing data and diaries, as well as direct non-medical costs via diaries, and informal care via questionnaire at proxy. Utility is examined as area under the curve.

A power calculation for our primary research question (checking the short-term effect of intensive rehabilitation versus standard care) yielded the following result: starting from a significance level of 0.05 and a standard deviation of our measurement of functionality of 20%, we will already find a significant difference between intensive rehabilitation and standard care of 12% with a group size of 90 patients and a power of 0.8. To control for dropout

we propose to recruit 100 patients so that we are fairly certain to have at least 90 patients for our primary analysis after three weeks.

The result corresponds to an effect size of 0.6, which is considered a 'medium' effect. However, we suspect that the effect will be even larger due to the clear difference in contrast of intensity of rehabilitation in the intensive group (30 hours of therapy per week) versus the control group (1-2 hours of therapy per week).

Drop-out is seen in scientific studies, however, we believe the probability in our study is lower than anticipated because of two reasons: (1) Measurements can all be done at home. So there is no demand on patients to move around; and (2) Patients in the control group receive an intensive rehabilitation programme between T3 and T4. This may just provide an incentive to stay in the study. But we will still include 11% more patients than necessary to accommodate any drop-out.

To be clear, no extra cost is charged to the patient. Patients who receive 3 weeks of intensive rehabilitation are not hospitalised, but can stay at Sporta near To Walk Again, if required.

--

DeepL

Subscribe to DeepL Pro to translate larger documents.
Visit www.DeepL.com/pro for more information.

Follow-up discussion RIZIV - To Walk Again (AZ Herentals)- KU Leuven
Wednesday 8 June 14-15 hrs.

As agreed, with this letter we are following up on the discussion that was held.

First of all, we would like to thank RIZIV for getting back in touch to further develop this project proposal.
This letter includes the following elements:

- Question and answer: brief description of the elements raised and response to them;
- Annex 1: Latest research protocol proposal, as submitted in 2021, with adjustments based on Q&A 8 June;
- Annex 2: Update of Excel document cost proposal.

Questions and answers

- Can more information be given about which therapy is given. This is quite unclear now and insufficient for reproducibility purposes.

It is true that the specific content of therapy (rehabilitation including high-technology therapy) is not described in detail. This will be further explained in the extended protocol, partly based on an international guideline, the TIDieR checklist[1]. The checklist will be further developed to be submitted as an appendix when the study protocol is published. An initial elaboration of this checklist is also attached to these submitted documents.
Obviously, the detailed elaboration is an element that will be coordinated by the trial manager, together with the trial therapist and To Walk Again (AZ Herentals). Rehabilitation is simply not a standard event because it addresses the specific problems for the patient and sets up its own exercise programme for that purpose.

- The study cited shows a lasting effect of a short, intensive rehabilitation up to 6 months, but what happens after that?

Indeed, based on the literature, judgements can only be made up to 6 months post intervention because this is the last measurement time point that was included. With our study, however, we then have a chance to check whether this is still the case at 9 months post-intervention. In itself, it is positive that individuals still have preservation of functioning documented months after the intervention. Indeed, the intervention includes self-management, which is learning skills to deal with problems experienced by oneself. This is regarded as one of the main reasons why months after the intervention ends, there is still preservation of progress in patients.

- The study includes a small target population (100 patients).

Comparing the number of patients in a clinical trial within rehabilitation to another domain such as pharmacology is not correct. The way an intervention is given, e.g. medication versus rehabilitation does not allow this comparison.
The basis for calculating the number of patients in a study is also (among other things) the effect expected and here the contrast in therapy plays a big role. For rehabilitation, this contrast in our study is going to be very large for amount of therapy (which is a crucial factor for effect), namely in the experimental group 90 hours in 3 weeks and in the control group 6 to 9 hours in three weeks. This large

[1] https://www.equator-network.org/reporting-guidelines/tidier/

contrast with the expected limited variation in effect allows significant and clinically relevant changes to be found in a limited group of patients.

- Are or can other rehabilitation centres involved?

Although multicentre studies are of benefit for generalisability of studied therapy, for rehabilitation studies they are also a source of scientific noise or bias, especially due to the rehabilitation provided. In one centre, this will be less variable due to the fact that fewer different therapists are involved in delivering the therapy (rehabilitation including high-technology therapy). This, in turn, is an advantage because it allows standardisation to be more rigorous. Of course, we continue to deal with individuals and their problems which will always result in some variability in therapy.

- What is the intention afterwards, after the project?

A successful outcome of the proposed study could lead to evidence-based material to move towards integration and recognition of the use of height technology in rehabilitation and of the added value of supporting continuing rehabilitation within the calibrated available funding instruments.

- The target group is CVA and spinal cord injury which is quite heterogeneous after all. Is this going to provide enough information? What about the time after onset of pathology and the person's condition before the onset of pathology? How will this be taken into account?

We are confident that the study will give meaningful results. Two pathologies may give more heterogeneity but limiting the study to 1 pathology is not going to directly lead to more homogeneity because within one pathology there is already clear heterogeneity. The more homogeneous the group is made, the less generalisable the results are. So it is searching for a balance. In itself, with the fact that we include individuals who have a rehabilitation profile, we have also already made a selection. We don't see the very mildly affected patients, because they don't have a chronic rehabilitation profile, and we don't see the very severely affected patients either, because they stay in a residential care centre or similar setting. So the study already includes a more homogeneous subpopulation of both pathologies.
The suggestion to include more specifically the time post pathology (and level of paraplegia) as well as the functional status of the patient before onset of pathology are valid additions and will be included in the protocol.

- The cost component should be made clear, in particular how the services can be proven so that RIZIV is certain that they were actually performed.

This can be done by sending an Excel list at calibrated times in which the various components (cf. figure 2 of the study protocol) are registered at patient level. Importantly, this is done anonymously because of GDPR. However, in case of an audit, this can be substantiated, among other things, by the registration that is going to happen in the context of the study. All performances will be documented in terms of time and content in the REDcap system that will be set up for this study. The latter is a requirement of the ethics committee.

TIDieR
Template for Intervention Description and Replication

The TIDieR (Template for Intervention Description and Replication) Checklist*:

Information to include when describing an intervention and the location of the information

Item number	Item	Where located ** Primary paper (page or appendix number)	Other (†) (details)
	**BRIEF NAME**		
**1.**	Provide the name or a phrase that describes the intervention.	Subtitle page 1 protocol	______________
	**WHY**		
**2.**	Describe any rationale, theory, or goal of the elements essential to the intervention.	Description and research framework page 1 and 2 protocol	______________
	**WHAT**		
**3.**	Materials: Describe any physical or informational materials used in the intervention, including those provided to participants or used in intervention delivery or in training of intervention providers. Provide information on where the materials can be accessed (e.g. online appendix, URL).	Page 6-9 protocol - further materials will be developed at the start of the project	______________
**4.**	Procedures: Describe each of the procedures, activities, and/or processes used in the intervention, including any enabling or supporting activities.	Page 6-9 protocol - further	______________

		material will be developed at the start of the project	
	**WHO PROVIDED**		
**5.**	For each category of intervention provider (e.g. psychologist, nursing assistant), describe their expertise, background and any specific training given.	Page 7 protocol	______________
	**HOW**		
**6.**	Describe the modes of delivery (e.g. face-to-face or by some other mechanism, such as internet or telephone) of the intervention and whether it was provided individually or in a group.	Page 6-9 protocol - further material will be developed at the start of the project	______________
	**WHERE**		
**7.**	Describe the type(s) of location(s) where the intervention occurred, including any necessary infrastructure or relevant features.	To Walk Again, Herentals (in protocol)	______________
	**WHEN and HOW MUCH**		
**8.**	Describe the number of times the intervention was delivered and over what period of time including the of sessions, their schedule, and their duration, intensity or dose.	Page 6-9 number protocol - further material is developed at the	______________

		start of the project	
	**TAILORING**		
**9.**	If the intervention was planned to be personalised, titrated or adapted, then describe what, why, when, and how.	Page 6-9 protocol - further material will be developed at the start of the project	______________
	**MODIFICATIONS**		
**10.‡**	If the intervention was modified during the course of the study, describe the changes (what, why, when, and how).	N/A, is noted in REDcap system	______________
	**HOW WELL**		
**11.**	Planned: If intervention adherence or fidelity was assessed, describe how and by whom, and if any strategies were used to maintain or improve fidelity, describe them.	N/A, will be elaborated in the context of process evaluation at project start	______________
**12.‡**	Actual: If intervention adherence or fidelity was assessed, describe the extent to which the intervention was delivered as planned.	N/A, will be elaborated in the context of process evaluation at project start	______________

** **Authors -** use N/A if an item is not applicable for the intervention being described. **Reviewers -** use '?' if information about the element is not reported/not sufficiently reported.

† If the information is not provided in the primary paper, give details of where this information is available. This may include locations such as a published protocol or other published papers (provide citation details) or a website (provide the URL).

ǂ If completing the TIDieR checklist for a protocol, these items are not relevant to the protocol and cannot be described until the study is complete.

* We strongly recommend using this checklist in conjunction with the TIDieR guide (see *BMJ* 2014;348:g1687) which contains an explanation and elaboration for each item.

* The focus of TIDieR is on reporting details of the intervention elements (and where relevant, comparison elements) of a study. Other elements and methodological features of studies are covered by other reporting statements and checklists and have not been duplicated as part of the TIDieR checklist. When a **randomised trial** is being reported, the TIDieR checklist should be used in conjunction with the CONSORT statement (see www.consort-statement.org) as an extension of **Item 5 of the CONSORT 2010 Statement.** When a **clinical trial protocol** is being reported, the TIDieR checklist should be used in conjunction with the SPIRIT statement as an extension of **Item 11 of the SPIRIT 2013 Statement** (see www.spirit-statement.org). For alternate study designs, TIDieR can be used in conjunction with the appropriate checklist for that study design (see www.equator-network.org).
